# AI literacy for self-regulated learning among pre-service teachers: development and validation of a scale of ethical use, monitoring, motivation, regulation, and evaluation

**DOI:** 10.3389/fpsyg.2026.1948694

**Published:** 2026-08-27

**Authors:** Ismet Kaya, Rusen Meylani

**Affiliations:** Ziya Gökalp Faculty of Education, Dicle University, Diyarbakır, Türkiye

**Keywords:** AI literacy, artificial intelligence, measurement invariance, pre-service teachers, scale development, self-regulated learning

## Abstract

**Introduction:**

Existing AI-literacy measures often emphasize broad knowledge, attitudes, application, or ethical awareness rather than how learners use AI within self-regulated academic learning. This study developed and validated the Multidimensional Aspects of AI Literacy Scale for Pre-Service Teachers (MAILS-PT), which assesses Ethical Use, Metacognitive Monitoring (Monitor), Motivational Regulation (Motivate), Behavioral Regulation (Regulate), and Critical Evaluation (Evaluate).

**Method:**

An initial 60-item pool was reduced to 40 items through expert content review. The scale was administered to 420 pre-service teachers recruited in Spring 2025 for exploratory factor analysis and to a separately recruited sample of 684 in Fall 2025 for confirmatory and validation analyses. Ordinal-data methods, reliability analysis, assessment of convergent and discriminant validity, and gender measurement-invariance testing were conducted. A 15-item AI-Assisted Learning Judgment Quiz was used to examine preliminary concurrent associations with a framework-aligned judgment measure.

**Results:**

Exploratory factor analysis retained 30 items, six per dimension; the five-factor solution explained 58.3% of the variance, with primary loadings ranging from 0.639–0.829. Confirmatory factor analysis supported the structure, *χ*^2^ (395) = 522.90, *p* < 0.001, CFI = 0.988, TLI = 0.986, RMSEA = 0.022, 90% CI [0.016, 0.027], SRMR = 0.037. Subscale omega values ranged from 0.851–0.889 in the exploratory sample and 0.848–0.874 in the confirmatory sample. AVE values ranged from 0.522–0.583 and HTMT values from 0.029–0.272. Configural, loading, and threshold invariance across gender were supported under the specific ordered-categorical JASP/lavaan parameterization. Ethical Use showed near-zero relationships with the other dimensions. The five subscales jointly explained 41.3% of judgment-quiz variance, *R* = 0.643, *R*^2^ = 0.413, adjusted *R*^2^ = 0.409, *F* (5, 678) = 95.48, *p* < 0.001.

**Conclusion:**

MAILS-PT provides preliminary psychometric support for five distinguishable aspects of learning-related AI literacy among pre-service teachers. Subscales should be interpreted separately, with Ethical Use treated as a distinct yet related domain of responsible academic AI use. Quiz associations provide preliminary framework-aligned concurrent evidence, not independent criterion or predictive validity. Further validation across institutions, cultures, and behavioral or longitudinal criteria is needed.

## Introduction

### Artificial intelligence and learning in higher education

Generative artificial intelligence has become increasingly embedded in higher education, where students use AI tools for writing, studying, explanation-seeking, brainstorming, receiving feedback, and academic decision-making (Chan and Hu, 2023; Chiu, 2024). Students value AI for personalized support, idea generation, writing assistance, and research while also expressing concerns about accuracy, privacy, ethics, and overreliance (Chan and Hu, 2023). Consequently, educationally meaningful AI use depends not only on access or frequency of use but also on how learners strategically, critically, and responsibly engage with AI during academic work (Jin et al., 2023; Molenaar et al., 2023).

AI-supported learning is therefore both a technological and a psychological process. Generative AI can provide explanations, examples, feedback, planning support, and revision suggestions, but these affordances alone do not constitute effective learning. Their educational value depends on learner agency, active engagement, judgment, and appropriate pedagogical conditions (Chiu, 2024; Jin et al., 2023; Molenaar et al., 2023). This distinction is particularly important for pre-service teachers because their capacity to use AI responsibly affects both their current academic learning and their future professional practice.

### Self-regulated learning in AI-assisted contexts

Self-regulated learning refers to learners’ active management of cognition, motivation, behavior, and learning strategies (Panadero, 2017). Its core processes include planning, monitoring, strategy selection, motivational regulation, reflection, and adaptation (Panadero, 2017; Molenaar et al., 2023). In AI-assisted contexts, learners may use generative AI to support these processes by identifying knowledge gaps, requesting alternative explanations, organizing tasks, sustaining engagement, evaluating outputs, and revising learning plans (Chiu, 2024; Jin et al., 2023).

However, learning-related AI literacy is not defined here as self-regulated learning performed in the presence of AI. It refers more narrowly to learners’ capacity to engage with AI appropriately as an external learning resource: knowing how to use AI to support regulatory processes while retaining learner agency, critically evaluating AI-generated information, and observing ethical boundaries governing academic AI use. Conventional self-regulated learning concerns how learners regulate learning generally; the present construct concerns how those regulatory and evaluative processes are enacted specifically through interaction with AI and its distinctive affordances and limitations.

Furthermore, this distinction is crucial for motivational regulation. AI may be used to make difficult material more approachable, generate alternative explanations, connect tasks to goals, or support persistence, but effective regulation remains under the learner’s control rather than being transferred to the system (Chiu, 2024; Jin et al., 2023). Accordingly, monitoring, motivation, and regulation are conceptualized here not as novel forms of self-regulated learning, but as AI-specific enactments of established regulatory processes.

### Ethical and critical AI use

AI-assisted learning also requires critical and ethical judgment. Generative AI can produce fluent academic text, explanations, and references, despite limitations in accuracy and provenance, making verification and critical evaluation important components of competent use (Long and Magerko, 2020; Mansoor et al., 2024). Learners must therefore evaluate claims, compare outputs with reliable sources, recognize unsupported or fabricated information, and determine whether particular uses of AI are appropriate for an academic task (Toker Gokce et al., 2024; Long and Magerko, 2020; Mansoor et al., 2024).

Ethical use is a related yet conceptually different domain. Academic uses of generative AI raise questions about authorship, disclosure, independent work, fairness, and compliance with course or institutional expectations (Lancaster, 2023; Perkins, 2023). Ethical AI use may therefore depend partly on normative and contextual factors rather than on the same psychological processes that underlie monitoring, motivation, or behavioral regulation. For this reason, Ethical Use is conceptualized in the present study as a distinct but related component of responsible AI-assisted learning rather than as evidence of a single homogeneous underlying disposition.

### Measurement gap

Existing AI-literacy measures make important contributions but typically assess broader competencies than the learning-regulatory processes targeted here. Recent instruments, for example, assess fundamental AI knowledge, impact assessment, performance evaluation, and practical application among higher-education students (Kang and Park, 2026); knowledge, operational, critical, and ethical dimensions among higher-education learners, including student teachers (Ranieri et al., 2025); and awareness, use, evaluation, and ethics among teachers (Gao and Wang, 2026).

These measures complement earlier conceptualizations that emphasize understanding, use, evaluation, and ethical engagement with AI (Long and Magerko, 2020; Toker Gokce et al., 2024; Mansoor et al., 2024). However, they do not specifically organize measurement around how learners use AI to monitor understanding, regulate motivation, organize learning behavior, critically evaluate AI-generated information, and maintain responsible academic conduct during learning. MAILS-PT therefore does not seek to replace general AI-literacy, digital-literacy, critical-information-literacy, or academic-integrity measures. Instead, it targets a narrower intersection between AI literacy and self-regulated academic learning.

Responsible AI use overlaps directly with the present framework in Ethical Use and Evaluate, but it is conceptually broader because it concerns appropriate AI practices across educational, professional, and societal contexts. Learning-related AI literacy, as defined here, is limited to strategic, regulatory, evaluative, and ethical engagement with AI during academic learning.

Compared with existing instruments, MAILS-PT addresses a different unit of measurement: it isolates how AI is incorporated into metacognitive monitoring, motivational regulation, behavioral regulation, and critical evaluation during the learning process, while keeping ethical academic AI use as a separately interpretable domain. Its contribution is therefore not broader coverage of AI literacy, but more specific measurement of the intersection between AI use and self-regulated academic learning.

This distinction matters because using AI to diagnose a knowledge gap, sustain engagement, revise a study plan, or verify a claim requires different judgments than merely knowing about AI, reporting frequent AI use, expressing favorable attitudes toward it, or possessing general digital competence (Chan and Hu, 2023; Jin et al., 2023; Lancaster, 2023; Perkins, 2023). The proposed measure consequently focuses on the quality and regulation of AI-assisted learning practices, rather than AI adoption or technical competence.

Scale-development research further emphasizes that score interpretations require multiple sources of evidence, including content evidence, internal structure, reliability, and relations with other variables (Boateng et al., 2018). Self-report scores alone cannot establish how learners judge realistic AI-related situations. The present study therefore also examined associations with a scenario-based judgment quiz. Because the quiz was developed within the same research program, follows the same five-domain framework, and was administered concurrently, it is treated only as a framework-aligned concurrent judgment measure, not as an independent behavioral criterion or evidence of predictive validity.

### Present study

The present study developed and evaluated the Multidimensional Aspects of AI Literacy Scale for Pre-Service Teachers (MAILS-PT). The instrument assesses five learning-related dimensions: Ethical Use, Metacognitive Monitoring (Monitor), Motivational Regulation (Motivate), Behavioral Regulation (Regulate), and Critical Evaluation (Evaluate). The abbreviated labels in parentheses are retained throughout the manuscript to maintain continuity with the terminology used in the originating project. It does not purport to measure the complete domain of AI literacy, including technical knowledge, computational understanding, programming, or AI-system design. Rather, it measures selected regulatory, evaluative, and ethical aspects of AI use during academic learning.

The study addressed the following research questions:

1. What factor structure characterizes MAILS-PT scores among pre-service teachers?
2. Do the resulting subscales demonstrate adequate internal consistency and convergent and discriminant validity?
3. Are the factor structure, factor loadings, and item thresholds invariant across gender?
4. How are the five dimensions related to one another, particularly given the expected relative independence of Ethical Use from the learning-regulation dimensions?
5. How are MAILS-PT subscale scores associated with performance on the concurrently administered, framework-aligned AI-Assisted Learning Judgment Quiz?

Monitor, Motivate, Regulate, and Evaluate were expected to show modest positive interrelations because they represent complementary processes in AI-assisted learning. Ethical Use was expected to be less strongly related because it concerns rule-governed academic responsibility and contextual norms rather than learning regulation itself.

## Theoretical framework

### Self-regulated learning as the core foundation

Self-regulated learning provides the primary theoretical foundation for MAILS-PT because it explains how learners manage cognition, motivation, behavior, and strategy use during academic work. Self-regulated learning involves planning, monitoring, selecting strategies, controlling motivation, reflecting, and adapting (Panadero, 2017; Molenaar et al., 2023). In AI-assisted learning, these processes may incorporate AI as an external resource for explanation, planning, feedback, and evaluation (Jin et al., 2023; Molenaar et al., 2023).

MAILS-PT does not treat these regulatory processes as uniquely AI-based competencies. Rather, it assesses how learners enact selected regulatory processes through interaction with AI while retaining responsibility for learning decisions. This distinction separates learning-related AI literacy from conventional self-regulated learning: the latter concerns regulation of learning generally, whereas the present framework concerns learners’ strategic, critical, and responsible use of AI within that regulation.

#### Metacognitive monitoring (monitor)

The Metacognitive Monitoring (Monitor) dimension concerns the metacognitive use of AI to assess what is understood, what remains unclear, and what requires further study. Learners may use AI to generate diagnostic questions, request alternative explanations, compare their understanding with expected knowledge, or identify conceptual gaps (Panadero, 2017; Jin et al., 2023).

Monitor therefore represents AI-supported metacognitive monitoring, not general metacognitive ability. The learner remains responsible for judging whether an AI-generated explanation has improved understanding and whether further learning is needed (Jin et al., 2023; Molenaar et al., 2023).

#### Motivational regulation (motivate)

Motivational regulation concerns learners’ efforts to sustain persistence, confidence, interest, and engagement during academic work (Panadero, 2017). Generative AI may support these processes by making difficult material more accessible, offering alternative explanations, connecting tasks with goals, or helping learners maintain engagement (Chiu, 2024).

The Motivational Regulation (Motivate) dimension therefore assesses the strategic use of AI for motivational regulation, rather than motivation toward AI itself or emotional dependence on AI. Learner agency remains central: AI serves as a resource for regulating motivation rather than as the source of that regulation (Chiu, 2024; Panadero, 2017).

#### Behavioral regulation (regulate)

Behavioral regulation includes setting goals, organizing tasks, managing time, sequencing activities, and revising ineffective plans (Panadero, 2017). AI can support these processes by helping learners break assignments into manageable steps, generate schedules, prioritize activities, or reconsider study plans (Jin et al., 2023).

The Behavioral Regulation (Regulate) dimension assesses AI-supported planning and behavioral regulation. It does not imply delegating control of learning to AI. Effective regulation requires learners to evaluate suggestions, make decisions, and revise their strategies (Panadero, 2017; Molenaar et al., 2023).

#### Critical evaluation (evaluate)

Critical evaluation is particularly important in generative-AI contexts because fluent outputs may still contain inaccurate, unsupported, or fabricated information. AI literacy therefore includes the capacity to evaluate AI-generated content rather than accept it uncritically (Long and Magerko, 2020; Mansoor et al., 2024).

The Critical Evaluation (Evaluate) dimension involves checking accuracy, comparing AI responses with reliable sources, identifying unsupported claims, and questioning outputs before using them. It represents critical and epistemically responsible engagement with AI-generated information (Long and Magerko, 2020; Perkins, 2023).

#### Ethical use

The Ethical Use dimension encompasses transparency, authorship, independent work, fairness, disclosure, and compliance with course or institutional expectations. These issues are central to the responsible academic use of generative AI (Lancaster, 2023; Perkins, 2023).

Ethical Use is theoretically different from the four learning-regulation dimensions. Monitor, Motivate, Regulate, and Evaluate address the processes directly involved in managing or evaluating learning with AI, whereas Ethical Use concerns the normative responsibility for how AI is used. Ethical judgments may therefore depend more strongly on course rules, institutional policies, disclosure expectations, and perceptions of academic responsibility than on learners’ regulatory strategies themselves.

Accordingly, Ethical Use is conceptualized as a distinct yet related subscale, rather than as evidence of a common underlying regulatory factor. Its inclusion reflects the view that responsible AI-assisted learning requires not only effective learning regulation but also appropriate academic conduct (Lancaster, 2023; Perkins, 2023).

### A multidimensional model of learning-related AI literacy

The framework therefore distinguishes five dimensions: Ethical Use, Metacognitive Monitoring (Monitor), Motivational Regulation (Motivate), Behavioral Regulation (Regulate), and Critical Evaluation (Evaluate). Monitor, Motivate, Regulate, and Evaluate were expected to show modest positive associations because they reflect distinct processes involved in AI-assisted learning. Ethical Use was expected to be comparatively independent because it concerns normative academic responsibility rather than learning regulation itself.

The model does not propose a single homogeneous AI-literacy trait. Instead, it conceptualizes learning-related AI literacy as a profile of distinct competencies and practices situated at the intersection of AI use, self-regulated learning, critical evaluation, and academic responsibility (Panadero, 2017; Jin et al., 2023; Long and Magerko, 2020; Perkins, 2023). Consequently, the five subscales are intended primarily for separate interpretation.

### Validity argument for MAILS-PT and the scenario-based quiz

MAILS-PT measures self-reported tendencies, whereas the AI-Assisted Learning Judgment Quiz assesses decisions in framework-aligned academic scenarios. Using different response formats allows examination of whether reported tendencies are associated with conceptually related judgment (Boateng et al., 2018).

However, the quiz was developed by the same research team, follows the same five-domain framework, and was administered concurrently with MAILS-PT. Associations between the two measures therefore provide only preliminary evidence of relations with a concurrently administered, framework-aligned judgment measure. They should not be interpreted as validation against an independent external criterion, as evidence of actual behavior, or evidence of predictive validity.

## Method

### Research design

This study used a multistage scale-development and validation design to develop the MAILS-PT. The process included item generation, content review, exploratory factor analysis (EFA), confirmatory factor analysis (CFA), reliability estimation, assessment of convergent and discriminant validity, gender measurement-invariance testing, and examination of concurrent associations with a framework-aligned judgment measure. Exploratory and confirmatory samples were recruited separately, consistent with recommendations for scale development (Boateng et al., 2018; Clark and Watson, 1995; Costello and Osborne, 2005; Worthington and Whittaker, 2006). The pilot phase was conducted in Spring 2025 and the main validation phase in Fall 2025.

Statistical analyses were conducted in JASP 0.96.0.0 (JASP Team, 2026). Exploratory procedures followed methods aligned with the *psych* framework (Revelle, 2018), and confirmatory analyses used structural equation modeling implemented in *lavaan* (Rosseel, 2012).

### Participants and recruitment

Participants were pre-service teachers enrolled in teacher-education programs at a public university in Türkiye. Eligibility required current enrollment in a program leading to a teaching qualification and prior experience with AI. Participants were recruited through announcements distributed to eligible students enrolled in teacher-education programs.

During the Spring 2025 semester, 450 pre-service teachers were invited to participate in the pilot phase, of whom 420 provided complete responses retained for analysis, corresponding to a response rate of 93.33%. For the main validation phase, 800 pre-service teachers were invited during the Fall 2025 semester, of whom 684 provided complete responses retained for analysis, corresponding to a response rate of 85.50%.

The pilot and main samples were recruited separately from different semester cohorts. The Spring 2025 cohort provided the exploratory-factor-analysis sample, whereas the Fall 2025 cohort provided the confirmatory and validation sample. Data collection was anonymous, and no identifying information was collected; consequently, participant identities could not be matched across the two administrations, and participant-level overlap was not formally assessed. The two samples are therefore described as separately recruited, temporally distinct semester-cohort samples rather than as samples with verified participant-level independence. Both samples were drawn from the same underlying institutional population of pre-service teachers enrolled in teacher-education programs. Their comparability was evaluated empirically rather than assumed. As reported in Table 1, the two samples did not differ significantly in age, gender, year of study, number of siblings, or marital status, and the corresponding effect sizes were negligible.

**Table 1:** Demographic characteristics of the pilot and main pre-service teacher samples.

Variable	Pilot (EFA; *n* = 420)	Main (CFA; *n* = 684)	Sample comparability
Age (years), *M* (SD)	24.79 (6.53)	24.88 (6.76)	*t* = −0.23, *p* = 0.818, *d* = 0.01
Gender, *n* (%)			***χ***^**2**^ **(1) = 0.40, *p* = 0.527, *V* = 0.02**
Male	172 (41.0)	267 (39.0)	
Female	248 (59.0)	417 (61.0)	
Year of study, *n* (%)			***χ***^**2**^ **(4) = 0.69, *p* = 0.952, *V* = 0.03**
1st year	50 (11.9)	76 (11.1)	
2nd year	218 (51.9)	366 (53.5)	
3rd year	80 (19.0)	135 (19.7)	
4th year	68 (16.2)	101 (14.8)	
Other	4 (1.0)	6 (0.9)	
Number of siblings, *n* (%)			***χ***^**2**^ **(5) = 0.69, *p* = 0.983, *V* = 0.03**
0	4 (1.0)	8 (1.2)	
1	23 (5.5)	34 (5.0)	
2	42 (10.0)	76 (11.1)	
3	60 (14.3)	102 (14.9)	
4	61 (14.5)	98 (14.3)	
5 or more	230 (54.8)	366 (53.5)	
Marital status, *n* (%)			***χ***^**2**^ **(1) = 0.22, *p* = 0.639, *V* = 0.01**
Single	365 (86.9)	601 (87.9)	
Married	55 (13.1)	83 (12.1)	

Percentages were calculated within each sample. Age was compared using Welch’s independent-samples *t*-test; categorical variables were compared using Pearson Chi-square tests without continuity correction. Cohen’s *d* and Cramér’s *V* are reported as effect sizes. Bold values indicate the overall Pearson chi-square test results for categorical variables.

Disciplinary specializations within teacher education were not collected. Detailed characteristics of prior AI experience—including frequency of use, prior AI training, type of generative-AI access, and awareness of institutional AI policies—were also not collected and therefore could not be examined as participant-level covariates.

### Procedure and data collection

Data were collected online in two temporally separated phases. The pilot administration was conducted in Spring 2025, and the main administration was conducted during Fall 2025. Participants first viewed an information and consent page and proceeded only after providing informed consent. Participation was voluntary, and responses were used solely for research purposes and analyzed in aggregate.

The pilot administration included the 40 MAILS-PT candidate items retained after content review. The main administration contained the same 40-item candidate scale, followed immediately by the 15-item AI-Assisted Learning Judgment Quiz, within the same online session. Only complete submissions were retained for analysis; consequently, the final analytic datasets contained no missing item-level responses. No completed submissions were excluded from either analytic sample; the difference between the number invited and the final sample size reflected nonparticipation rather than post-response data exclusion. No separate attention-check, response-time, or duplicate-response exclusion criteria were applied because no completed submissions were excluded from the analytic samples. Because responses were anonymous and no identifying information was collected, possible participation in both semester administrations could not be assessed at the participant level.

### Instrument development and content validation

The initial pool contained 60 items, with 12 items for each of five dimensions: Ethical Use, Monitor, Motivate, Regulate, and Evaluate. Items were first written in English and then translated into Turkish. The Turkish version was independently back-translated into English, and the original and back-translated versions were compared for conceptual equivalence. Discrepancies were resolved by the research team with attention to construct consistency, clarity, and natural Turkish expression. Responses were recorded on a five-point Likert-type scale ranging from 1 = *strongly disagree* to 5 = *strongly agree*.

Content review involved the two authors, both assistant professors in educational sciences, and four external experts selected based on relevant disciplinary expertise: two associate professors of educational psychology, one associate professor with language expertise, and one assistant professor of educational technology.

All six reviewers rated item relevance on a four-point scale and evaluated clarity, wording, and construct alignment. Because the two authors had participated in item development, the content-validity indices were treated as preliminary evidence from combined internal and external review rather than as ratings from six independent experts. Item-level content-validity indices were calculated as the proportion of reviewers who assigned ratings of 3 or 4. Review of relevance, clarity, construct alignment, and redundancy reduced the pool from 60 to 40 items, with eight candidate items retained per dimension.

No cognitive interviews, think-aloud procedures, or formal target-user response-process studies were conducted before administration. Accordingly, the study provides content-based evidence from expert review but not direct response-process evidence; this limitation is addressed in the Discussion.

### AI-assisted learning judgment quiz

A 15-item scenario-based AI-Assisted Learning Judgment Quiz was developed to examine associations between self-reported tendencies and judgment in AI-supported academic contexts. The quiz included three multiple-choice items for each of the five domains: Ethical Use, Monitor, Motivate, Regulate, and Evaluate.

Correct responses were scored 1 and incorrect responses 0, yielding total scores from 0 to 15. McDonald’s omega was 0.715, and Cronbach’s alpha was 0.704. Because the quiz was developed by the same researchers, followed the same five-domain framework, contained only three items per domain, and was administered immediately after MAILS-PT, it was treated as a preliminary, concurrently administered, framework-aligned judgment measure rather than as an independent external criterion or a validated standalone instrument.

### Scoring

MAILS-PT items are rated on a 5-point Likert-type scale ranging from 1 = strongly disagree to 5 = strongly agree. Subscale scores are calculated as the mean of the six retained items for each dimension: Ethical Use (a1, a4–a8), Monitor (b1–b6), Motivate (c1, c3, c4, c6–c8), Regulate (d1–d6), and Evaluate (e1–e4, e7, e8). Higher scores indicate stronger self-reported endorsement of the behaviors represented by that specific dimension.

Primary interpretation should be based on the five subscale scores rather than a total score because the instrument is multidimensional and Ethical Use is relatively independent of the learning-regulation dimensions. An overall mean was calculated solely for descriptive purposes and should not be interpreted as a homogeneous scale score. Because only complete submissions were analyzed, no missing-item scoring procedure was required for the present analyses.

### Exploratory factor analysis

EFA was conducted on the pilot sample using a polychoric correlation matrix because the items used five ordered response categories (Holgado-Tello et al., 2010). Factors were extracted via principal axis factoring with Promax rotation. Principal axis factoring was selected to estimate latent dimensions from shared variance, and an oblique rotation was appropriate because correlations among dimensions were theoretically permissible.

Sampling adequacy and factorability were evaluated using the Kaiser–Meyer–Olkin measure and Bartlett’s test of sphericity. Factor retention was informed jointly by parallel analysis, scree-plot inspection, and theoretical interpretability. Parallel analysis was conducted using principal-component eigenvalues because this was the implementation available in the JASP procedure used for factor retention, whereas the substantive factor solution was estimated using principal axis factoring to model shared variance among latent dimensions. The component-based parallel analysis was therefore treated only as an empirical retention heuristic and was not used as the factor-extraction model. The decision to retain five factors was based on convergence among parallel analysis, scree-plot inspection, theoretical interpretability, and the resulting principal-axis-factor solution rather than on component eigenvalues alone.

Item-retention decisions were based on primary loadings, uniqueness, cross-loading patterns, redundancy, and conceptual consistency. The pre-specified numerical criteria were a primary loading of at least 0.55, uniqueness below 0.60, and a difference of at least 0.20 between the two largest factor loadings. These criteria were selected to favor items that clearly represent their intended latent dimension and have limited item-specific variance. Items failing one or more statistical or conceptual criteria were removed, producing the final 30-item solution.

### Confirmatory factor analysis

CFA was conducted on the separately recruited main sample to evaluate the five-factor structure identified through EFA. The model specified five correlated latent variables—Ethical Use, Monitor, Motivate, Regulate, and Evaluate—with the 30 retained items loading on their intended factors. Because the indicators were ordinal, the model was estimated using diagonally weighted least squares (DWLS). Model fit was evaluated using *χ*^2^, CFI, TLI, RMSEA, SRMR, GFI, MFI, RNI, and NFI, with interpretation emphasizing approximate fit indices because *χ*^2^ is sensitive to sample size.

Modification indices were not examined. No correlated residuals, cross-loadings, item deletions, or other post-hoc model modifications were introduced after estimation. The reported confirmatory model therefore represents the pre-specified five-factor structure derived from the exploratory analysis rather than a model subsequently adjusted to improve fit.

### Reliability and construct validity

Internal consistency was evaluated using McDonald’s omega and Cronbach’s alpha. Omega was emphasized because it is less sensitive than alpha to equal factor loadings, whereas alpha was retained for comparability with previous research (Dunn et al., 2014).

Convergent validity was evaluated using the average variance extracted (AVE), with values above 0.50 considered adequate. Discriminant validity was examined using heterotrait–monotrait ratios (HTMT). Because Ethical Use was theoretically expected to be comparatively independent, its low correlations with the other dimensions were interpreted in relation to both discriminant validity and its distinct conceptual status.

### Gender measurement invariance

Gender measurement invariance was examined using multi-group confirmatory factor analysis for ordered categorical indicators in JASP, using structural-equation-modeling implemented through lavaan. All 30 retained indicators were specified as ordinal, and the models were estimated using diagonally weighted least squares (DWLS). The same five-factor structure was specified across gender groups.

The invariance models followed the sequence implemented in the JASP/lavaan procedure used in the present study: configural invariance, loading invariance, and threshold invariance. In the configural model, the same five-factor structure was specified for male and female participants without equality constraints on corresponding factor loadings. In the loading-invariance model, corresponding factor loadings were constrained to be equal across groups. In the subsequent threshold-invariance model, the loading constraints were retained, and corresponding item thresholds were additionally constrained to equality across groups. Because alternative approaches to measurement invariance with ordered categorical indicators may assess threshold equality before loading equality, the present sequence should be understood as the specific ordered-categorical parameterization implemented by the JASP/lavaan procedure used in this study rather than as a universal sequence for ordinal invariance testing.

Invariance was evaluated primarily by changes in approximate fit. Changes of no more than 0.010 in CFI and 0.015 in RMSEA were interpreted as supporting invariance across successive models (Chen, 2007; Putnick and Bornstein, 2016). Model *χ*^2^, degrees of freedom, CFI, TLI, RMSEA with 90% confidence intervals, and SRMR were also examined. Because the identification requirements changed when threshold equality constraints were introduced, the number of freely estimated parameters and latent-mean constraints were also inspected directly in the model outputs to verify that the non-monotonic degrees-of-freedom sequence reflected the implemented model identification rather than a specification or reporting error.

### Concurrent associations with scenario-based judgment

Multiple linear regression was used to examine concurrent associations between the five MAILS-PT subscale scores and total performance on the AI-Assisted Learning Judgment Quiz. Ethical Use, Monitor, Motivate, Regulate, and Evaluate were entered simultaneously as explanatory variables. Tolerance, variance inflation factors, and condition indices were examined to assess multicollinearity.

Because the scale and quiz were developed within the same research program, followed the same conceptual framework, and were administered concurrently, these analyses were interpreted as preliminary associations with a framework-aligned judgment measure, not as independent criterion validity, behavioral prediction, or prospective validity.

### Descriptive analyses

Descriptive statistics were calculated separately for the pilot and main samples. Welch’s independent-samples *t* tests and Cohen’s *d* were used to compare subscale scores across samples. After measurement-invariance testing, observed subscale means were also summarized by gender. These comparisons were descriptive; no latent-mean model was estimated.

## Results

### Sample comparability

The pilot EFA sample included 420 pre-service teachers, and the main CFA sample included 684. As shown in Table 1, the samples were demographically comparable.

The mean age was 24.79 years (SD = 6.53) in the pilot sample and 24.88 years (SD = 6.76) in the main sample. The difference was not significant, Welch’s *t* = −0.23, *p* = 0.818, *d* = 0.01.

Gender distributions were also similar, *χ*^2^ (1) = 0.40, *p* = 0.527, Cramér’s *V* = 0.02. Males accounted for 41.0% of the pilot sample and 39.0% of the main sample; females accounted for 59.0 and 61.0%, respectively.

No significant differences were found for year of study, *χ*^2^ (4) = 0.69, *p* = 0.952, Cramér’s *V* = 0.03; number of siblings, *χ*^2^ (5) = 0.69, *p* = 0.983, Cramér’s *V* = 0.03; or marital status, *χ*^2^ (1) = 0.22, *p* = 0.639, Cramér’s *V* = 0.01. All observed between-sample differences were nonsignificant and the corresponding effect sizes were negligible, indicating that the two separately recruited semester cohorts were demographically comparable on the characteristics measured in this study.

The samples represented different semester cohorts recruited in Spring and Fall 2025, respectively, and their similarity on the measured demographic characteristics was examined empirically rather than presumed from their common institutional setting. Because responses were anonymous and no identifying information was collected, participant-level overlap across the two administrations could not be formally assessed.

### Content validity and item screening

Content validity was evaluated using ratings from two research-team members and four external experts. Full item-level ratings are reported in Appendix A and item-selection decisions are summarized in Appendix B. Each item was rated on a four-point relevance scale, and item-level content validity indices were calculated as the proportion of raters assigning scores of 3 or 4.

Across the initial 60-item pool, S-CVI values ranged from 0.67 to 0.76 across the five dimensions. After review for relevance, conceptual clarity, construct alignment, and redundancy, 40 items were selected for EFA, with eight items per dimension. For the selected items, S-CVI values increased to 0.85–0.92. Regulate showed the highest selected-item S-CVI (0.92), followed by Ethical Use, Motivate, and Evaluate (0.90 each), and Monitor (0.85).

Most selected items had I-CVI values between 0.83 and 1.00. After EFA, 30 items were retained, comprising six items for each of the five dimensions. Table 2 summarizes the item-reduction process and construct-level S-CVI values.

**Table 2:** Content validity and item retention across the five aspects of AI literacy.

Construct	Initial items	Selected by expert opinion	Retained after EFA	S-CVI, all items	S-CVI, selected items for EFA
Ethical use	12	8	6	0.71	0.90
Monitor	12	8	6	0.67	0.85
Motivate	12	8	6	0.71	0.90
Regulate	12	8	6	0.74	0.92
Evaluate	12	8	6	0.76	0.90
Total	60	40	30	—	—

I-CVI, item-level content validity index; S-CVI, scale-level content validity index; EFA, exploratory factor analysis. S-CVI values for all items refer to the original 12-item pool within each construct. S-CVI values for selected items refer to the eight items per construct retained after expert review and submitted to EFA. S-CVI values were calculated using ratings from two members of the research team and four external experts. Therefore, they should not be interpreted as estimates based exclusively on independent external raters. S-CVI refers to the average of the item-level CVI values within each construct.

### Exploratory factor analysis

An exploratory factor analysis was conducted with the pilot sample (*N* = 420) using principal axis factoring with Promax rotation.

#### Preliminary analyses

Sampling adequacy was strong. The overall Kaiser–Meyer–Olkin value was 0.853, and individual MSA values ranged from 0.795 to 0.905. Bartlett’s test of sphericity was significant, *χ*^2^ (435) = 6982.267, *p* < 0.001, confirming factorability.

Mardia’s multivariate skewness test was nonsignificant, *χ*^2^ (4960) = 4897.287, *p* = 0.734, as was the small-sample corrected statistic, *χ*^2^ (4960) = 4934.536, *p* = 0.598. Multivariate kurtosis was significant, *z* = −5.990, *p* < 0.001. Given the ordinal response format and the use of principal axis factoring, this departure was not considered problematic.

#### Factor retention

Parallel analysis supported retaining five factors. The first five observed eigenvalues—5.994, 4.221, 3.402, 3.021, and 2.906—exceeded their corresponding simulated mean eigenvalues, whereas the sixth observed eigenvalue (0.783) did not exceed the simulated value (1.269). The parallel-analysis results are reported in Table 3.

**Table 3:** Parallel analysis supporting the five-factor solution.

Factor	Real data component eigenvalue	Simulated data mean eigenvalue
Factor 1*	5.994	1.542
Factor 2*	4.221	1.470
Factor 3*	3.402	1.405
Factor 4*	3.021	1.355
Factor 5*	2.906	1.308
Factor 6	0.783	1.269

*Factor should be retained. Parallel analysis was based on principal-component eigenvalues as implemented in JASP, whereas the substantive EFA was estimated using principal axis factoring with Promax rotation. The component-based analysis served only as one factor-retention criterion and was not used to estimate the final factor solution.

**Figure 1:**
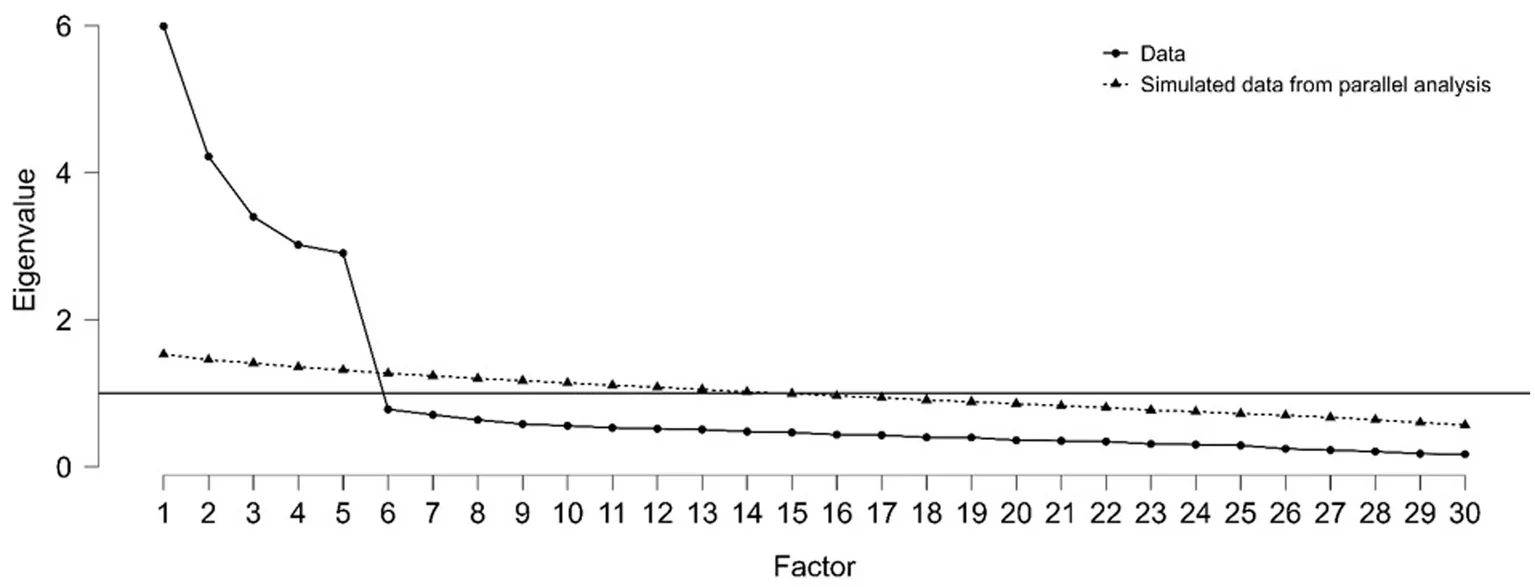
Scree plot and parallel analysis for the pilot sample.

The scree plot also showed a clear decline through the fifth factor, with flattening from the sixth factor onward, providing further support for the five-factor solution (Figure 1).

The rotated five-factor solution accounted for 58.3% of the total variance. Factor-specific eigenvalues, proportions of variance, and cumulative variance are reported in Table 4.

**Table 4:** Eigenvalues and variance explained for the five-factor EFA solution.

Factor	Eigenvalue	Proportion var.	Cumulative	SumSq. loadings	Proportion var.	Cumulative
Factor 1	5.574	0.186	0.186	3.743	0.125	0.125
Factor 2	3.836	0.128	0.314	3.716	0.124	0.249
Factor 3	2.992	0.100	0.413	3.402	0.113	0.362
Factor 4	2.597	0.087	0.500	3.383	0.113	0.475
Factor 5	2.490	0.083	0.583	3.245	0.108	0.583

Extraction method = principal axis factoring. Rotation = Promax.

#### Item retention and factor loadings

Item retention was based on three pre-specified statistical criteria together with conceptual interpretability: a primary factor loading of at least 0.55, uniqueness below 0.60, and a difference of at least 0.20 between the two largest factor loadings. These relatively conservative criteria were selected to retain items that showed a clear association with their intended latent dimension, limited item-specific variance, and adequate empirical separation from competing factors. The criteria were considered alongside conceptual interpretability rather than applied as isolated mechanical cutoffs.

Of the 40 candidate items entered into EFA, 10 were removed: a2, a3, b7, b8, c2, c5, d7, d8, e5, and e6. All 10 removed items had uniqueness values exceeding 0.60, indicating that an unacceptably large proportion of their variance was not accounted for by the common-factor solution. None of the removed items showed problematic cross-loadings, and the retained items also met the pre-specified loading-separation criterion. The remaining 30 items satisfied the statistical criteria and showed conceptually interpretable factor membership. The resulting solution contained six items per dimension; equal subscale length was not itself an item-retention criterion.

- Evaluate: e1–e4, e7-e8.
- Ethical use: a1, a4–a8.
- Regulate: d1–d6.
- Monitor: b1–b6.
- Motivate: c1, c3-c4, c6–c8.

Primary loadings ranged from 0.639 to 0.829, and uniqueness values ranged from 0.323 to 0.565. Evaluate loadings ranged from 0.675 to 0.829, Ethical Use from 0.752 to 0.808, Regulate from 0.646 to 0.807, Monitor from 0.704 to 0.804, and Motivate from 0.639 to 0.817.

The retained standardized loadings and uniqueness values are presented in Table 5. The complete unsuppressed pattern matrix, including all secondary loadings, is provided in Appendix D.

**Table 5:** Standardized EFA pattern loadings and uniqueness values for the retained items.

Item	Factor 1	Factor 2	Factor 3	Factor 4	Factor 5	Uniqueness
e1	0.792					0.372
e2	0.819					0.327
e3	0.817					0.364
e4	0.675					0.482
e7	0.760					0.414
e8	0.829					0.323
a1		0.796				0.367
a4		0.808				0.336
a5		0.786				0.379
a6		0.767				0.404
a7		0.752				0.426
a8		0.794				0.375
d1			0.792			0.398
d2			0.702			0.481
d3			0.807			0.348
d4			0.787			0.417
d5			0.646			0.508
d6			0.757			0.434
b1				0.804		0.347
b2				0.762		0.400
b3				0.704		0.488
b4				0.720		0.490
b5				0.716		0.466
b6				0.783		0.406
c1					0.725	0.452
c3					0.639	0.565
c4					0.817	0.361
c6					0.682	0.500
c7					0.817	0.344
c8					0.693	0.536

Loadings below 0.55 were suppressed. Factors were extracted using principal axis factoring with Promax rotation. Uniqueness values, rather than communalities, are reported. Factor 1 = Evaluate; Factor 2 = Ethical Use; Factor 3 = Regulate; Factor 4 = Monitor; Factor 5 = Motivate.

#### Factor correlations

Factor correlations ranged from −0.065 to 0.281. The strongest association was between Evaluate and Regulate (*r* = 0.281), followed by Evaluate and Motivate (*r* = 0.220), Monitor and Motivate (*r* = 0.206), and Regulate and Monitor (*r* = 0.199).

Ethical Use showed near-zero or slightly negative correlations with the other dimensions, indicating substantial empirical separation from the four learning-regulation and evaluation dimensions. The conceptual implications of this pattern are discussed in the Discussion.

The full correlation matrix is shown in Table 6.

**Table 6:** Correlations among the five EFA factors.

Factor	Factor 1	Factor 2	Factor 3	Factor 4	Factor 5
Factor 1	1.000				
Factor 2	−0.055	1.000			
Factor 3	0.281	0.013	1.000		
Factor 4	0.163	0.056	0.199	1.000	
Factor 5	0.220	−0.065	0.192	0.206	1.000

Factor 1 = Evaluate; Factor 2 = Ethical Use; Factor 3 = Regulate; Factor 4 = Monitor; Factor 5 = Motivate.

### Confirmatory factor analysis

A confirmatory factor analysis was conducted on the main sample (*n* = 684) to test the five-factor structure identified in the EFA. The model specified five correlated latent variables—Ethical Use, Monitor, Motivate, Regulate, and Evaluate—with the 30 retained items loading onto their intended factors. Because the items were ordinal, the model was estimated using diagonally weighted least squares.

#### Model fit

The five-factor model yielded fit indices consistent with the proposed structure, with *χ*^2^ (395) = 522.90, *p* < 0.001, CFI = 0.988, TLI = 0.986, RMSEA = 0.022, 90% CI [0.016, 0.027], and SRMR = 0.037. Additional indices included GFI = 0.992, MFI = 0.997, RNI = 0.988, and NFI = 0.952. No correlated residuals, cross-loadings, item deletions, or other post-hoc model modifications were introduced.

Full fit statistics are reported in Table 7.

**Table 7:** Fit indices for the five-factor confirmatory factor model.

Index	Value
*χ* ^2^	522.90
d*f*	395
*p*	<0.001
CFI	0.988
TLI	0.986
NFI	0.952
RNI	0.988
RMSEA	0.022
RMSEA 90% CI	[0.016, 0.027]
SRMR	0.037
GFI	0.992
MFI	0.997

Estimator = DWLS. Fit indices are scaled because the model included categorical indicators.

**Figure 2:**
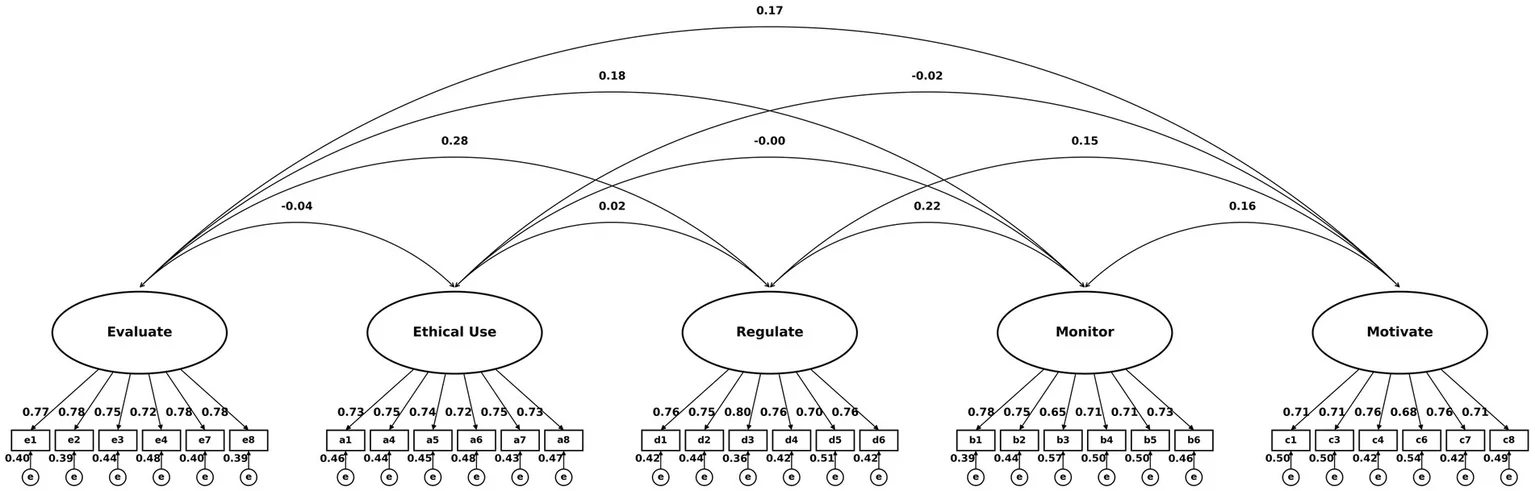
Five-factor confirmatory model of the multidimensional aspects of AI literacy scale for pre-service teachers. The model includes five correlated dimensions: ethical use, monitor, motivate, regulate, and evaluate. values on the single-headed paths are standardized factor loadings, values on the curved double-headed paths are standardized latent-factor correlations, and values associated with the item residuals represent unexplained variance.

The standardized measurement model is presented in Figure 2.

#### Standardized factor loadings

All freely estimated loadings were statistically significant (*p* < 0.001). Standardized loadings ranged from 0.653 to 0.802, indicating moderate-to-strong relations between the retained items and their intended latent factors.

Evaluate loadings ranged from 0.723 to 0.782; Ethical Use from 0.719 to 0.754; Regulate from 0.702 to 0.802; Monitor from 0.653 to 0.782; and Motivate from 0.681 to 0.762. The full unstandardized and standardized loading estimates are reported in Table 8.

**Table 8:** Unstandardized and standardized CFA factor loadings.

Factor	Indicator	Unstd. estimate	SE	*z*	*p*	95% CI	Std. loading *λ*
Evaluate	e1	1.000	0.000	—	—	[1.000, 1.000]	0.772
	e2	1.008	0.042	24.15	<0.001	[0.927, 1.090]	0.778
	e3	0.969	0.040	24.43	<0.001	[0.891, 1.047]	0.748
	e4	0.937	0.042	22.19	<0.001	[0.854, 1.020]	0.723
	e7	1.005	0.044	22.75	<0.001	[0.919, 1.092]	0.776
	e8	1.013	0.039	26.26	<0.001	[0.937, 1.089]	0.782
Ethical use	a1	1.000	0.000	—	—	[1.000, 1.000]	0.731
	a4	1.021	0.042	24.10	<0.001	[0.938, 1.104]	0.746
	a5	1.015	0.042	24.35	<0.001	[0.933, 1.097]	0.742
	a6	0.984	0.041	23.84	<0.001	[0.903, 1.065]	0.719
	a7	1.031	0.039	26.59	<0.001	[0.955, 1.107]	0.754
	a8	0.996	0.040	25.06	<0.001	[0.918, 1.074]	0.728
Regulate	d1	1.000	0.000	—	—	[1.000, 1.000]	0.763
	d2	0.977	0.047	20.95	<0.001	[0.886, 1.069]	0.746
	d3	1.051	0.041	25.68	<0.001	[0.971, 1.131]	0.802
	d4	1.001	0.040	25.08	<0.001	[0.923, 1.079]	0.764
	d5	0.920	0.048	19.22	<0.001	[0.827, 1.014]	0.702
	d6	0.999	0.044	22.68	<0.001	[0.913, 1.085]	0.762
Monitor	b1	1.000	0.000	—	—	[1.000, 1.000]	0.782
	b2	0.960	0.040	23.99	<0.001	[0.882, 1.039]	0.751
	b3	0.835	0.040	20.68	<0.001	[0.755, 0.914]	0.653
	b4	0.903	0.039	22.98	<0.001	[0.826, 0.980]	0.706
	b5	0.905	0.042	21.33	<0.001	[0.822, 0.988]	0.708
	b6	0.935	0.039	24.12	<0.001	[0.859, 1.011]	0.731
Motivate	c1	1.000	0.000	—	—	[1.000, 1.000]	0.709
	c3	1.000	0.056	17.83	<0.001	[0.890, 1.110]	0.710
	c4	1.074	0.052	20.64	<0.001	[0.972, 1.176]	0.762
	c6	0.960	0.056	17.21	<0.001	[0.851, 1.069]	0.681
	c7	1.070	0.050	21.34	<0.001	[0.972, 1.169]	0.760
	c8	1.002	0.049	20.25	<0.001	[0.905, 1.099]	0.711

Unstandardized loadings are reported using marker-variable identification, with the first loading fixed to 1.000 within each factor. Standardized loadings were obtained from the standardized CFA solution. All freely estimated loadings were statistically significant, *p* < 0.001.

### Factor variances and covariances

All latent factor variances were statistically significant (*p*s < 0.001), indicating meaningful variability in each construct. Latent covariances were generally small to modest. Evaluate was positively associated with Regulate, Monitor, and Motivate, and Regulate was positively associated with Monitor and Motivate. Ethical Use was not significantly associated with any other factors, supporting its empirical distinctiveness. The complete latent variance and covariance estimates are presented in Table 9.

**Table 9:** Latent factor variances and covariances in the confirmatory factor model.

Panel A: latent factor variances						
Factor	Estimate	SE	*z*	*p*	95% CI lower	95% CI upper
Evaluate	0.596	0.036	16.510	<0.001	0.525	0.667
Ethical use	0.535	0.035	15.480	<0.001	0.467	0.602
Regulate	0.582	0.036	16.320	<0.001	0.512	0.652
Monitor	0.612	0.031	19.830	<0.001	0.551	0.672
Motivate	0.503	0.040	12.450	<0.001	0.424	0.583

Panel B: latent factor covariances						
Factor pair	Estimate	SE	*z*	*p*	95% CI lower	95% CI upper
Evaluate ↔ Ethical use	−0.025	0.025	−1.014	0.311	−0.073	0.023
Evaluate ↔ Regulate	0.163	0.025	6.508	<0.001	0.114	0.212
Evaluate ↔ Monitor	0.106	0.026	4.047	<0.001	0.055	0.157
Evaluate ↔ Motivate	0.093	0.024	3.807	<0.001	0.045	0.140
Ethical use ↔ Regulate	0.011	0.025	0.425	0.671	−0.038	0.059
Ethical use ↔ Monitor	−0.001	0.025	−0.033	0.973	−0.051	0.049
Ethical use ↔ Motivate	−0.012	0.022	−0.552	0.581	−0.056	0.031
Regulate ↔ Monitor	0.132	0.026	5.052	<0.001	0.081	0.183
Regulate ↔ Motivate	0.083	0.024	3.429	<0.001	0.036	0.131
Monitor ↔ Motivate	0.086	0.025	3.451	<0.001	0.037	0.135

Estimates are unstandardized parameters from the CFA solution. All latent factor variances in Panel A were statistically significant, *p*s < 0.001. Covariances in Panel B are unstandardized latent-factor covariance estimates.

#### Convergent and discriminant validity

AVE values ranged from 0.522 to 0.583, exceeding the conventional 0.50 threshold for all five factors, thereby supporting convergent validity. Factor-specific AVE values are reported in Table 10.

**Table 10:** Average variance extracted for the five AI-literacy dimensions.

Factor	AVE
Evaluate	0.583
Ethical use	0.543
Regulate	0.573
Monitor	0.523
Motivate	0.522

AVE, average variance extracted.

HTMT values ranged from 0.029 to 0.272 and were below conventional cutoffs, providing evidence that the five dimensions were empirically distinguishable. The especially low values for Ethical Use were consistent with its marked empirical separation from the other four dimensions and were therefore not interpreted solely as favorable discriminant-validity evidence.

The full HTMT matrix is presented in Table 11.

**Table 11:** Heterotrait–Monotrait ratios among the five AI-literacy dimensions.

Factor	Evaluate	Ethical use	Regulate	Monitor	Motivate
Evaluate	1.000				
Ethical use	0.041	1.000			
Regulate	0.272	0.041	1.000		
Monitor	0.148	0.040	0.213	1.000	
Motivate	0.140	0.029	0.126	0.124	1.000

Values below the diagonal are HTMT ratios.

### Reliability analysis

Internal consistency was assessed separately in the EFA and CFA samples using McDonald’s omega and Cronbach’s alpha. All five subscales showed strong reliability across both samples.

In the EFA sample, omega values ranged from 0.851 to 0.889, and alpha values ranged from 0.851 to 0.881. In the CFA sample, omega values ranged from 0.848 to 0.874, and alpha values ranged from 0.846 to 0.868.

The highest EFA omega was observed for Ethical Use, *ω* = 0.889, whereas the highest CFA omega was observed for Evaluate, *ω* = 0.874. Full reliability estimates for Ethical Use, Monitor, Motivate, Regulate, and Evaluate are presented in Table 12.

**Table 12:** Internal consistency estimates across the EFA and CFA samples.

Construct	EFA *ω*	EFA *α*	CFA *ω*	CFA *α*
Evaluate	0.888	0.881	0.874	0.868
Ethical use	0.889	0.879	0.857	0.851
Regulate	0.868	0.861	0.873	0.867
Monitor	0.865	0.863	0.848	0.846
Motivate	0.851	0.851	0.848	0.847
Total scale	0.915	0.829	0.901	0.817

EFA, exploratory factor analysis sample; CFA, confirmatory factor analysis sample; *ω*, McDonald’s omega; *α*, Cronbach’s alpha. Overall 30-item coefficients are reported descriptively and do not establish that the scale is unidimensional. Primary interpretation should be based on the five subscale scores.

Overall 30-item coefficients were also calculated for descriptive purposes: *ω* = 0.915 and *α* = 0.829 in the EFA sample, and *ω* = 0.901 and *α* = 0.817 in the CFA sample. These total-scale coefficients should not be taken as evidence of unidimensionality or as justification for a single total score. The five-factor structure and the near-zero associations between Ethical Use and the other dimensions support interpreting the five subscale scores separately.

### Measurement invariance across gender

Measurement invariance across gender was examined using multi-group confirmatory factor analysis with diagonally weighted least squares (DWLS) estimation for ordered categorical indicators. All 30 retained items were specified as ordinal. The invariance models followed the sequence implemented in the JASP/lavaan procedure used in the present study: configural invariance, loading invariance, and threshold invariance. In the configural model, the same five-factor structure was specified for male and female participants while factor loadings and item thresholds were permitted to vary according to the model identification requirements. In the loading-invariance model, corresponding factor loadings were constrained to be equal across gender groups. In the subsequent threshold-invariance model, equality constraints were additionally imposed on corresponding item thresholds. This sequence is reported explicitly because, for ordered categorical indicators, some ordinal-invariance approaches test threshold equality before loading equality; the present analysis instead reflects the parameterization implemented by the JASP/lavaan procedure used for these models.

Model fit is summarized in Table 13 and is detailed as follows:

- The configural model showed good fit to the data, *χ*^2^ (790) = 912.82, *p* = 0.002, CFI = 0.988, TLI = 0.986, RMSEA = 0.021, 90% CI [0.014, 0.027], and SRMR = 0.047. These results supported the same five-factor configuration across male and female participants.
- The loading-invariance model also showed good fit, *χ*^2^ (815) = 927.61, *p* = 0.004, CFI = 0.989, TLI = 0.988, RMSEA = 0.020, 90% CI [0.012, 0.026], and SRMR = 0.048. Relative to the configural model, CFI increased by 0.001, and RMSEA decreased by 0.001, yielding ΔCFI = +0.001 and ΔRMSEA = −0.001. Both changes were well within the pre-specified criteria of |ΔCFI| ≤ 0.010 and |ΔRMSEA| ≤ 0.015, indicating that constraining corresponding factor loadings to equality across gender did not produce meaningful deterioration in model fit. The results therefore supported loading invariance.
- The threshold-invariance model likewise showed good fit, *χ*^2^ (810) = 922.26, *p* = 0.004, CFI = 0.989, TLI = 0.988, RMSEA = 0.020, 90% CI [0.012, 0.026], and SRMR = 0.048. Relative to the loading-invariance model, CFI and RMSEA were unchanged at the reported precision, yielding ΔCFI = 0.000 and ΔRMSEA = 0.000. Thus, adding equality constraints on corresponding item thresholds did not meaningfully reduce model fit, supporting threshold invariance across gender.

**Table 13:** Fit indices and changes across gender measurement-invariance models.

Model	*χ* ^2^	d*f*	*p*	CFI	TLI	RMSEA	RMSEA 90% CI	SRMR	ΔCFI	ΔRMSEA
Configural	912.82	790	0.002	0.988	0.986	0.021	[0.014, 0.027]	0.047	—	—
Loading invariance	927.61	815	0.004	0.989	0.988	0.020	[0.012, 0.026]	0.048	+0.001	−0.001
Threshold invariance	922.26	810	0.004	0.989	0.988	0.020	[0.012, 0.026]	0.048	0.000	0.000

Estimator = DWLS for ordered categorical indicators. ΔCFI and ΔRMSEA compare each model with the immediately preceding model. Configural invariance specifies the same five-factor structure across gender groups without equality constraints on corresponding factor loadings. Loading invariance constrains corresponding factor loadings to equality across groups. Threshold invariance retains the loading constraints and additionally constrains corresponding item thresholds to equality across groups. Changes of |ΔCFI| ≤ 0.010 and |ΔRMSEA| ≤ 0.015 were interpreted as supporting invariance (Chen, 2007; Putnick and Bornstein, 2016).

The decrease in degrees of freedom from the loading-invariance model (d*f* = 815), to the threshold-invariance model (d*f* = 810), reflects the identification and parameterization used by the ordered-categorical JASP/lavaan model rather than a reversal of model constraint. The loading-invariance output contained 295 freely estimated parameters, whereas the threshold-invariance output contained 300. In the loading-invariance model, latent factor means were fixed to zero in both groups. In the threshold-invariance model, the five latent factor means in the non-reference group were released, whereas the corresponding means in the reference group remained fixed for identification. The five additional freely estimated latent means therefore account for the five-degree-of-freedom reduction. The model outputs directly document the change from 295 to 300 free parameters as well as the corresponding treatment of the latent factor means.

Taken together, the approximate fit indices remained highly stable across the three models, and the invariance conclusion was based primarily on changes in approximate fit rather than on the monotonic ordering of model degrees of freedom. Changes in CFI and RMSEA were substantially smaller than the pre-specified criteria, supporting configural, loading, and threshold invariance across gender under the specific ordered-categorical parameterization implemented in the JASP/lavaan analysis. The results therefore indicate that, within this parameterization, the proposed five-factor structure, corresponding factor loadings, and item thresholds were comparable across male and female pre-service teachers. Measurement invariance should not, however, be interpreted as evidence that the groups have equal levels of the underlying constructs. Accordingly, the observed gender-specific subscale means reported below are presented descriptively rather than as formal latent-mean comparisons.

### Gender-stratified results

After establishing support for loading and threshold invariance, subscale means were examined descriptively by gender. As shown in Table 14, scores were broadly similar across male and female pre-service teachers in both samples.

**Table 14:** Subscale means by gender across the pilot and main samples.

Scale/Factor	Pilot male *M* (SD)	Pilot female *M* (SD)	Main male *M* (SD)	Main female *M* (SD)
Ethical use	3.55 (0.83)	3.43 (0.85)	3.57 (0.81)	3.40 (0.84)
Monitor	3.21 (0.94)	3.25 (0.92)	3.22 (0.87)	3.22 (0.89)
Motivate	3.46 (0.87)	3.46 (0.81)	3.41 (0.83)	3.41 (0.85)
Regulate	3.26 (0.81)	3.25 (0.85)	3.16 (0.84)	3.21 (0.86)
Evaluate	3.53 (0.92)	3.54 (0.85)	3.54 (0.87)	3.51 (0.83)
Overall	3.40 (0.48)	3.38 (0.44)	3.38 (0.45)	3.35 (0.44)

Pilot male *n* = 172; pilot female *n* = 248; main male *n* = 267; main female *n* = 417. Scores were calculated using retained items only.

In the pilot sample, males reported slightly higher Ethical Use scores, whereas females reported slightly higher Monitor and Evaluate scores. Motivate and Regulate scores were nearly identical across gender groups. In the main sample, males again reported somewhat higher Ethical Use scores, while the remaining subscale means were nearly equivalent.

Across both samples, Evaluate and Ethical Use tended to have the highest means, whereas Monitor and Regulate tended to be lower. Because these comparisons were descriptive, they should not be interpreted as formal tests of latent mean differences. No latent mean model was estimated; therefore, the reported values reflect observed subscale scores only.

Full gender-stratified means and standard deviations are reported in Table 14.

### Concurrent associations with scenario-based judgment

Concurrent associations between MAILS-PT scores and performance on the 15-item AI-Assisted Learning Judgment Quiz were examined using multiple linear regression. The quiz total score was the dependent variable, and Ethical Use, Monitor, Motivate, Regulate, and Evaluate were entered simultaneously as explanatory variables.

The overall model was statistically significant, *F* (5, 678) = 95.48, *p* < 0.001, with *R* = 0.643, *R*^2^ = 0.413, adjusted *R*^2^ = 0.409, and RMSE = 2.486. Thus, the five MAILS-PT dimensions jointly explained 41.3% of the variance in concurrently administered judgment-quiz scores.

All five dimensions showed statistically significant positive unique associations with quiz performance. Motivate had the largest standardized association, *β* = 0.284, followed by Ethical Use, *β* = 0.273; Monitor, *β* = 0.235; Regulate, *β* = 0.227; and Evaluate, *β* = 0.222. All coefficients were statistically significant at *p* < 0.001. The complete regression coefficients and collinearity statistics are reported in Table 15.

**Table 15:** Multiple regression predicting scenario-based judgment scores from MAILS-PT dimensions.

Predictor	*B*	SE	*β*	*t*	*p*	Tolerance	VIF
Intercept	−9.034	0.744	—	−12.15	<0.001	—	—
Ethical use	1.061	0.114	0.273	9.27	<0.001	0.998	1.002
Monitor	0.863	0.111	0.235	7.78	<0.001	0.946	1.057
Motivate	1.089	0.115	0.284	9.49	<0.001	0.964	1.037
Regulate	0.859	0.117	0.227	7.36	<0.001	0.914	1.094
Evaluate	0.845	0.117	0.222	7.23	<0.001	0.922	1.084

*N* = 684; *R* = 0.643; *R*^2^ = 0.413; adjusted *R*^2^ = 0.409; RMSE = 2.486, *F* (5, 678) = 95.48, *p* < 0.001. Predictors were entered simultaneously. The largest condition index was 20.82. Ethical Use = a_avg; Monitor = b_avg; Motivate = c_avg; Regulate = d_avg; Evaluate = e_avg.

Multicollinearity was not evident. Tolerance values ranged from 0.914 to 0.998, and VIF values ranged from 1.002 to 1.094. The largest condition index was 20.82. Taken together, these diagnostics indicated that the unique regression coefficients were not adversely affected by problematic collinearity across the five MAILS-PT dimensions.

The fitted regression equation was:


$$\begin{array}{l} \text{Quiz score}=-9.034+1.061(\text{ethical}\;\mathrm{use})+0.863(\text{monitor})+\\ 1.089(\text{motivate})+0.859(\text{regulate})+\\ 0.845(\text{evaluate}) \end{array}$$

These coefficients reflect conditional concurrent associations rather than causal effects or prospective prediction. Because MAILS-PT and the judgment quiz were developed within the same research program, share the same five-domain conceptual framework, and were administered sequentially within the same session, the observed associations should be interpreted as preliminary framework-aligned concurrent evidence rather than independent criterion validity, behavioral prediction, or prospective validity.

#### Quiz item difficulty and discrimination

Item difficulty and discrimination indices for the 15-item scenario-based quiz are reported in Table 16. Difficulty values ranged from 0.167 to 0.569. Thirteen items were of moderate difficulty, Item 12 was difficult, and Item 2 was very difficult. Corrected item–total correlations ranged from 0.015 to 0.547.

**Table 16:** Difficulty and discrimination indices for the scenario-based judgment items.

Item	Difficulty (*p*)	Difficulty classification	Corrected item–total *r*	Discrimination classification
Item 1	0.461	Moderate	0.274	Acceptable
Item 2	0.167	Very difficult	0.015	Weak
Item 3	0.523	Moderate	0.547	Very good
Item 4	0.423	Moderate	0.414	Very good
Item 5	0.497	Moderate	0.128	Weak
Item 6	0.414	Moderate	0.355	Good
Item 7	0.529	Moderate	0.415	Very good
Item 8	0.510	Moderate	0.473	Very good
Item 9	0.459	Moderate	0.226	Acceptable
Item 10	0.404	Moderate	0.084	Weak
Item 11	0.535	Moderate	0.534	Very good
Item 12	0.348	Difficult	0.287	Acceptable
Item 13	0.569	Moderate	0.198	Weak
Item 14	0.494	Moderate	0.365	Good
Item 15	0.519	Moderate	0.284	Acceptable

*N* = 684; Item difficulty represents the proportion of participants who answered each item correctly; higher values indicate easier items. Item discrimination was estimated using the corrected point-biserial correlation between each dichotomously scored item and the quiz total excluding that item. Difficulty values below 0.20 were classified as very difficult, values from 0.20 to 0.39 as difficult, values from 0.40 to 0.59 as moderate, values from 0.60 to 0.79 as easy, and values of 0.80 or higher as very easy. Corrected item–total correlations below 0.20 were classified as weak, values from 0.20 to 0.29 as acceptable, values from 0.30 to 0.39 as good, and values of 0.40 or higher as very good.

Items 3, 4, 7, 8, and 11 showed very good discrimination; Items 6 and 14 showed good discrimination; Items 1, 9, 12, and 15 showed acceptable discrimination; and Items 2, 5, 10, and 13 showed weak discrimination.

Most items showed acceptable difficulty and discrimination; however, Items 2, 5, 10, and 13 showed weak discrimination, and Item 2 was both very difficult and nearly unrelated to the remaining quiz score. These weaknesses limit interpretation of the quiz total score and support treating it as a preliminary framework-aligned judgment measure rather than a validated standalone instrument. Items 2, 5, 10, and 13 should be revised and reevaluated in future applications.

### Construct means across EFA and CFA samples

Construct scores were calculated using retained items only. As shown in Table 17, mean scores were highly similar across the pilot EFA and main CFA samples.

**Table 17:** Subscale scores across the pilot and main samples.

Scale/Factor	Pilot EFA *M* (SD)	Main CFA *M* (SD)	Test statistic
Ethical use	3.48 (0.85)	3.47 (0.83)	*t* = 0.28, *p* = 0.782, *d* = 0.02
Monitor	3.23 (0.93)	3.22 (0.88)	*t* = 0.12, *p* = 0.901, *d* = 0.01
Motivate	3.46 (0.83)	3.41 (0.84)	*t* = 1.00, *p* = 0.316, *d* = 0.06
Regulate	3.25 (0.83)	3.19 (0.85)	*t* = 1.20, *p* = 0.230, *d* = 0.07
Evaluate	3.53 (0.88)	3.52 (0.85)	*t* = 0.24, *p* = 0.812, *d* = 0.01
Overall	3.39 (0.46)	3.36 (0.45)	*t* = 1.06, *p* = 0.289, *d* = 0.07

Construct scores were calculated using retained items only. EFA sample size was *n* = 420; CFA sample size was *n* = 684. Welch’s *t* tests were used. Positive *d* values indicate higher means in the EFA sample.

Welch’s t tests showed no significant between samples for Ethical Use, *t* = 0.28, *p* = 0.782, *d* = 0.02; Monitor, *t* = 0.12, *p* = 0.901, *d* = 0.01; Motivate, *t* = 1.00, *p* = 0.316, *d* = 0.06; Regulate, *t* = 1.20, *p* = 0.230, *d* = 0.07; or Evaluate, *t* = 0.24, *p* = 0.812, *d* = 0.01.

The descriptive overall mean was also similar across samples, with 3.39 (SD = 0.46) in the EFA sample and 3.36 (SD = 0.45) in the CFA sample, *t* = 1.06, *p* = 0.289, *d* = 0.07.

All effect sizes were negligible to very small. Because MAILS-PT is multidimensional and Ethical Use was largely independent of the other dimensions, the overall mean should not be interpreted as a unitary scale score.

### Item-level descriptive statistics

Item-level descriptive statistics for the retained items are presented in Table 18. Item means were stable across the pilot EFA and main CFA samples.

**Table 18:** Item means and standard deviations across the pilot and main samples.

Item	Pilot EFA *M* (SD)	Main CFA *M* (SD)
Ethical use		
a1	3.69 (1.00)	3.69 (1.06)
a4	3.84 (0.88)	3.83 (0.94)
a5	3.50 (1.08)	3.48 (1.10)
a6	3.38 (1.11)	3.36 (1.12)
a7	3.30 (1.17)	3.29 (1.18)
a8	3.18 (1.15)	3.16 (1.18)
Monitor		
b1	2.89 (1.17)	2.89 (1.15)
b2	3.60 (1.15)	3.61 (1.12)
b3	3.33 (1.21)	3.34 (1.16)
b4	3.12 (1.22)	3.08 (1.19)
b5	3.37 (1.22)	3.34 (1.20)
b6	3.07 (1.24)	3.07 (1.21)
Motivate		
c1	3.87 (1.07)	3.77 (1.10)
c3	3.57 (1.10)	3.52 (1.12)
c4	3.20 (1.12)	3.14 (1.12)
c6	3.80 (1.03)	3.74 (1.08)
c7	3.21 (1.14)	3.18 (1.14)
c8	3.10 (1.12)	3.10 (1.16)
Regulate		
d1	3.25 (1.08)	3.18 (1.11)
d2	3.26 (1.06)	3.22 (1.09)
d3	3.07 (1.17)	2.98 (1.18)
d4	2.95 (1.17)	2.89 (1.19)
d5	3.37 (1.00)	3.35 (1.02)
d6	3.62 (1.02)	3.53 (1.02)
Evaluate		
e1	3.54 (1.12)	3.56 (1.10)
e2	3.57 (1.13)	3.55 (1.10)
e3	3.27 (1.12)	3.25 (1.11)
e4	3.78 (1.09)	3.71 (1.07)
e7	3.83 (1.00)	3.84 (0.97)
e8	3.20 (1.21)	3.20 (1.18)

Descriptive statistics were calculated using retained items only. EFA sample size was *n* = 420; CFA sample size was *n* = 684.

In the pilot sample, item means ranged from 2.89 to 3.87; in the main sample, they ranged from 2.89 to 3.84. The highest mean in the pilot sample was observed for c1, *M* = 3.87, SD = 1.07, whereas the highest mean in the main sample was for e7, *M* = 3.84, SD = 0.97. The lowest mean in the pilot sample was for b1, *M* = 2.89, SD = 1.17, and the lowest means in the main sample were for b1, *M* = 2.89, SD = 1.15, and d4, *M* = 2.89, SD = 1.19.

Standard deviations ranged from 0.88 to 1.24 in the pilot sample and from 0.94 to 1.21 in the main sample, indicating adequate response variability. Overall, the retained items showed consistent descriptive patterns and substantial response variability across samples.

## Discussion

### Overview and measurement contribution

This study developed MAILS-PT as a multidimensional measure of learning-related AI literacy among pre-service teachers. The findings provided support for five distinguishable dimensions—Ethical Use, Monitor, Motivate, Regulate, and Evaluate—across separately recruited exploratory and confirmatory samples. Evidence was obtained from content review, internal structure, reliability, convergent and discriminant validity, gender measurement invariance, and associations with a concurrently administered scenario-based judgment measure. This approach aligns with contemporary views of validation as an accumulating argument for score interpretation rather than a single statistical test (American Educational Research Association et al., 2014; Boateng et al., 2018; Kane, 2013; Messick, 1995). The present results support dimension-specific interpretation, not a single homogeneous AI-literacy score.

MAILS-PT differs from broader AI-literacy instruments by focusing specifically on how learners use AI in monitoring, motivational regulation, behavioral regulation, critical evaluation, and responsible academic conduct. Recent measures appropriately assess broader AI knowledge, application, evaluation, and ethical competencies (Gao and Wang, 2026; Kang and Park, 2026; Ranieri et al., 2025), whereas MAILS-PT targets the narrower intersection of AI literacy and self-regulated academic learning.

### Interpretation of the five dimensions

Monitor, Motivate, and Regulate represent AI-supported enactments of established self-regulated learning processes rather than new forms of self-regulation. Their interpretation aligns with research emphasizing metacognitive monitoring, motivational control, planning, strategic action, and adaptation (Chiu, 2024; Jin et al., 2023; Molenaar et al., 2023; Panadero, 2017). Evaluate adds a specifically AI-relevant epistemic requirement: learners must judge the accuracy and credibility of generated information rather than treat fluent output as inherently reliable (Long and Magerko, 2020; Mansoor et al., 2024).

The modest correlations among these four dimensions are theoretically plausible rather than problematic. Monitoring understanding, sustaining motivation, regulating study behavior, and evaluating information are complementary but functionally distinct processes. Their modest associations therefore support a profile interpretation in which learners may be relatively strong in one aspect of AI-assisted learning while weaker in another. The results do not support treating these dimensions as interchangeable manifestations of one general regulatory tendency.

### The distinct status of ethical use

Ethical Use requires particular attention. It showed near-zero correlations with the other dimensions in EFA and nonsignificant latent covariances with them in CFA. This pattern should not be interpreted merely as unusually strong discriminant validity. Instead, it suggests that ethical academic AI use may operate through mechanisms substantially different from those underlying learning regulation.

Several explanations are plausible. Ethical conduct may depend strongly on course rules, institutional policies, disclosure expectations, and perceived academic-integrity norms (Lancaster, 2023; Perkins, 2023). Responses may also be more susceptible to socially desirable responding because ethical items concern explicitly normative behavior. Furthermore, the wording and theoretical basis of Ethical Use emphasize rule-governed responsibility, whereas Monitor, Motivate, Regulate, and Evaluate concern learning or epistemic processes. These differences may help explain its empirical independence.

Accordingly, Ethical Use is best retained as a distinct but related subscale within MAILS-PT rather than interpreted as evidence for a common higher-order construct. Its inclusion reflects the view that responsible AI-assisted academic learning involves both effective learning practices and appropriate conduct, while the empirical results require these aspects to remain separate in score interpretation.

### Construct validity and gender invariance

AVE and HTMT results indicated that the dimensions were empirically distinguishable, but the particularly low HTMT values involving Ethical Use should be interpreted together with its broader theoretical separation rather than solely as favorable discriminant-validity evidence (Fornell and Larcker, 1981; Henseler et al., 2015).

Configural, loading, and threshold invariance were supported across gender under the ordered-categorical parameterization implemented in the JASP/lavaan analysis. The stability of CFI and RMSEA across successive models indicated that constraining corresponding factor loadings and subsequently item thresholds did not meaningfully reduce model fit. The findings therefore support comparability of the five-factor measurement structure, factor loadings, and ordered-category thresholds across male and female participants (Chen, 2007; Putnick and Bornstein, 2016).

The non-monotonic degrees-of-freedom sequence reflects the identification changes associated with the ordered-categorical model. When threshold equality constraints were imposed, five latent factor means in the non-reference group were freely estimated, accounting for the corresponding reduction in degrees of freedom. This pattern is consistent with established treatments of identification in multi-group confirmatory factor analysis with ordered categorical indicators (Millsap and Yun-Tein, 2004; Wu and Estabrook, 2016). Accordingly, the invariance conclusion is specific to the ordered-categorical JASP/lavaan parameterization used here. It should not be interpreted as establishing equivalence across all alternative identification sequences for ordinal measurement-invariance testing.

The gender-stratified results do not contradict measurement invariance. Observed means were very similar across gender for Monitor, Motivate, Regulate, and Evaluate, while males showed somewhat higher Ethical Use scores in both samples. Because no latent-mean comparison was conducted, these differences remain descriptive and should not be interpreted as formal evidence of gender differences.

### Associations with scenario-based judgment

All five MAILS-PT dimensions were positively associated with performance on the AI-Assisted Learning Judgment Quiz. These findings show correspondence between self-reported tendencies and decisions within conceptually aligned scenarios, extending the validity argument beyond internal structure alone (American Educational Research Association et al., 2014; Boateng et al., 2018; Kane, 2013).

However, this evidence is necessarily preliminary. The quiz was developed by the same researchers, followed the same five-domain framework, and was administered immediately after MAILS-PT. It therefore does not constitute an independent external criterion. Its modest reliability and weak discrimination for several items further limit interpretation of the total score. The associations should consequently be described as relations with a concurrently administered, framework-aligned judgment measure rather than behavioral, predictive, or independent criterion validity. The quiz should not presently be regarded as a validated standalone instrument.

### Theoretical, measurement, and practical contributions

The theoretical contribution of MAILS-PT is to distinguish AI-supported regulatory processes from self-regulated learning itself: the scale assesses how established regulatory processes are enacted through interaction with AI, together with critical evaluation and ethical responsibility. Its measurement contribution is the provision of five separately interpretable subscales supported across separately recruited exploratory and confirmatory samples.

Its practical contribution should be stated more cautiously. MAILS-PT may currently be used for research, formative exploration, and program-level evaluation. Subscale profiles may help researchers or educators identify areas that warrant further investigation or instructional attention. However, a low individual score should not itself be interpreted as evidence that intervention is required.

MAILS-PT should not presently be used for misconduct allegations, disciplinary decisions, admissions, employment screening, certification, or other high-stakes individual classifications. Cut scores, classification accuracy, predictive validity, and consequences of such uses have not been established. A total score should likewise not replace interpretation of the five subscales, particularly given the independence of Ethical Use.

### Limitations and future research

Several limitations constrain interpretation. First, both samples were drawn from pre-service teachers at a single public university in Türkiye. The use of separately recruited Spring and Fall semester samples provided temporal separation between the exploratory and confirmatory analyses, but participant-level independence could not be formally verified because data collection was anonymous and no identifying information was collected. The samples also do not provide geographic, institutional, or national replication. Future studies should examine the structure and invariance of MAILS-PT across institutions, teacher-education programs, countries, and cultural settings.

Second, MAILS-PT is self-reported and cross-sectional. Scores may therefore be influenced by response styles and social desirability and cannot establish actual AI behavior, causal relationships, or prospective outcomes. Future studies should incorporate independently obtained behavioral indicators, AI-use records, academic products, instructor ratings, longitudinal outcomes, or learning-performance measures.

Third, prior AI use was required for participation, but detailed information on the frequency of AI use, access to generative AI, prior AI training, and awareness of institutional AI policies was not collected. Disciplinary specialization was also not recorded. These factors may influence how participants interpret and respond to AI-related items and should be examined in future validation studies.

Fourth, content review included appropriately qualified internal and external reviewers, but cognitive interviews, think-aloud procedures, and formal target-user response-process studies were not conducted. Consequently, the study provides content evidence from expert judgment but limited direct evidence concerning how respondents cognitively interpreted the items.

Fifth, the scenario-based quiz was brief, demonstrated modest internal consistency, and contained several weakly discriminating items. Because it was developed within the same conceptual framework and administered immediately after MAILS-PT, shared content and administration-order effects may have contributed to the observed associations. Future research should revise the weak items and test MAILS-PT against independently developed, behaviorally grounded, and longitudinal criteria.

Finally, the near-zero relationships involving Ethical Use require replication. Future studies should examine whether this pattern reflects a genuinely separate ethical domain, institutional policy dependence, socially desirable responding, item framing, or differences in how ethical expectations are understood across educational contexts.

## Conclusion

This study developed and validated the MAILS-PT to assess five learning-related aspects of AI use: Ethical Use, Metacognitive Monitoring (Monitor), Motivational Regulation (Motivate), Behavioral Regulation (Regulate), and Critical Evaluation (Evaluate). The scale was developed from an initial 60-item pool, refined through expert review, explored in a separately recruited pilot sample of 420 pre-service teachers, and then confirmed in a separately recruited sample of 684 participants. The final 30-item instrument showed a stable five-factor structure, strong subscale reliability, adequate convergent validity, clear empirical separation among dimensions, and configural, loading, and threshold invariance across gender under the specific ordered-categorical parameterization implemented in the JASP/lavaan analysis.

The findings support interpreting MAILS-PT as a multidimensional profile rather than a single homogeneous AI-literacy score. Monitor, Motivate, Regulate, and Evaluate represent distinct yet related ways learners engage with AI in self-regulated learning, whereas Ethical Use appears substantially more independent and is best treated as a distinct but related domain of responsible academic AI use. This distinction is theoretically important and should guide score interpretation.

MAILS-PT scores were also positively associated with performance on a concurrently administered, framework-aligned scenario-based judgment measure. These associations provide preliminary evidence that self-reported tendencies align with related AI-supported academic judgments, but they should not be interpreted as evidence of independent criterion validity, behavioral prediction, or prospective validity because the quiz was developed within the same research framework, administered immediately after the scale, and exhibited modest psychometric limitations.

The study has several limitations. Both samples were drawn from a single Turkish university; the design was cross-sectional and self-reported; detailed characteristics of prior AI experience were not collected; response-process evidence from cognitive interviews was unavailable; and the scenario-based quiz requires further refinement. Accordingly, MAILS-PT should currently be used primarily for research, formative assessment, and program-level evaluation, not for high-stakes individual decisions.

Overall, MAILS-PT offers preliminary psychometric support for assessing how pre-service teachers engage AI strategically, critically, and responsibly during academic learning. Further validation across institutions, disciplines, cultures, policy contexts, and with independently obtained behavioral or longitudinal criteria is needed before broader or higher-stakes interpretations are warranted.

## Data Availability

The datasets presented in this study can be found in online repositories. The names of the repository/repositories and accession number(s) can be found below: DOI: 10.5281/zenodo.21578499.
