# Supplementary material for: AI literacy for self-regulated learning among pre-service teachers: development and validation of a scale of ethical use, monitoring, motivation, regulation, and evaluation
Source: Front Psychol. 2026 Aug 27;17:1948694. doi: 10.3389/fpsyg.2026.1948694 (PMC13558209; doi:10.3389/fpsyg.2026.1948694)
Supplement: Supplementary file 1 [file Supplementary_file_1.pdf]

APPENDICES

Appendix A

Content-Validity Ratings, Item-Level CVI Values, and Item-Retention Decisions

Construct	Survey Item Code	Auth. 1 Score	Auth. 2 Score	Exp. 1 Score	Exp. 2 Score	Exp. 3 Score	Exp.4 Score	Average	I-CVI	S-CVI (All Items)	S-CVI (Selected Items for EFA)	Selected by expert opinion	Retained after EFA
Ethical Use	a01	3	3	4	3	3	3	3.17	1.00	0.71	0.90	Yes	Yes
	a02	3	3	3	4	4	3	3.33	1.00			Yes	No
	a03	3	4	4	4	3	4	3.67	1.00			Yes	No
	a04	4	3	4	3	2	2	3.00	0.67			Yes	Yes
	a05	4	4	4	4	4	2	3.67	0.83			Yes	Yes
	a06	3	3	3	4	4	4	3.50	1.00			Yes	Yes
	a07	4	3	3	4	4	2	3.33	0.83			Yes	Yes
	a08	3	3	3	3	4	2	3.00	0.83			Yes	Yes
	a09	3	3	3	1	3	1	2.33	0.67			No	
	a10	2	1	1	2	1	3	1.67	0.17			No	
	a11	3	3	1	2	2	2	2.17	0.33			No	
	a12	3	1	2	2	1	1	1.67	0.17			No	
Monitor	b01	4	3	3	3	2	4	3.17	0.83	0.67	0.85	Yes	Yes
	b02	3	4	4	3	3	2	3.17	0.83			Yes	Yes
	b03	3	4	4	3	4	2	3.33	0.83			Yes	Yes
	b04	3	3	3	3	2	3	2.83	0.83			Yes	Yes
	b05	3	4	4	4	2	3	3.33	0.83			Yes	Yes
	b06	3	4	4	4	2	4	3.50	0.83			Yes	Yes
	b07	4	3	4	4	3	2	3.33	0.83			Yes	No
	b08	3	4	4	3	3	3	3.33	1.00			Yes	No
	b09	2	2	2	2	3	1	2.00	0.17			No	
	b10	3	3	3	2	2	3	2.67	0.67			No	
	b11	2	1	2	2	1	2	1.67	0.00			No	
	b12	3	1	3	2	2	2	2.17	0.33			No	
Motivate	c01	4	3	4	4	2	4	3.50	0.83	0.71	0.90	Yes	Yes
	c02	3	3	3	3	2	2	2.67	0.67			Yes	No
	c03	4	3	4	4	4	3	3.67	1.00			Yes	Yes
	c04	3	3	3	3	3	4	3.17	1.00			Yes	Yes
	c05	3	4	3	3	2	3	3.00	0.83			Yes	No
	c06	3	4	4	4	3	3	3.50	1.00			Yes	Yes
	c07	3	3	3	3	2	3	2.83	0.83			Yes	Yes
	c08	4	3	3	4	4	3	3.50	1.00			Yes	Yes
	c09	1	1	2	2	3	3	2.00	0.33			No	
	c10	1	1	2	1	3	1	1.50	0.17			No	
	c11	3	3	2	1	1	2	2.00	0.33			No	
	c12	2	3	3	1	3	2	2.33	0.50			No	
Regulate	d01	3	3	4	3	3	2	3.00	0.83	0.74	0.92	Yes	Yes
	d02	3	4	4	3	4	4	3.67	1.00			Yes	Yes
	d03	3	3	3	3	3	3	3.00	1.00			Yes	Yes
	d04	4	4	3	4	4	2	3.50	0.83			Yes	Yes
	d05	4	4	3	4	4	3	3.67	1.00			Yes	Yes
	d06	3	4	3	4	4	3	3.50	1.00			Yes	Yes
	d07	3	4	3	3	4	2	3.17	0.83			Yes	No
	d08	4	4	4	3	2	3	3.33	0.83			Yes	No
	d09	2	1	3	3	3	1	2.17	0.50			No	
	d10	2	2	3	1	1	3	2.00	0.33			No	
	d11	3	3	3	2	2	1	2.33	0.50			No	
	d12	1	1	1	3	1	1	1.33	0.17			No	
Evaluate	e01	3	4	3	3	4	3	3.33	1.00	0.76	0.90	Yes	Yes
	e02	4	4	3	4	3	3	3.50	1.00			Yes	Yes
	e03	4	3	4	4	2	4	3.50	0.83			Yes	Yes
	e04	3	3	4	4	4	4	3.67	1.00			Yes	Yes
	e05	3	4	3	3	3	2	3.00	0.83			Yes	No
	e06	3	4	4	3	3	2	3.17	0.83			Yes	No
	e07	4	4	3	4	3	4	3.67	1.00			Yes	Yes
	e08	3	3	3	3	2	2	2.67	0.67			Yes	Yes
	e09	3	3	1	2	3	3	2.50	0.67			No	
	e10	2	1	1	3	2	3	2.00	0.33			No	
	e11	2	3	3	1	3	2	2.33	0.50			No	
	e12	3	3	1	1	3	2	2.17	0.50			No	

Appendix B

Initial Item Pool and Item-Retention Decisions for the Multidimensional Aspects of AI Literacy Scale for Pre-Service Teachers

Construct	Item code	English	Turkish	Selected by expert opinion	Retained after EFA
**Ethical Use**	**a01**	I avoid submitting AI-generated work as if it were entirely my own.	Yapay zekâ ile oluşturulmuş bir çalışmayı tamamen kendi emeğimmiş gibi sunmaktan kaçınırım.	Yes	Yes
	**a02**	I acknowledge the use of AI tools when my instructor requires it.	Öğretmenim sorduğunda yapay zekâ kullandığımı açıkça belirtirim.	Yes	No
	**a03**	I consider whether my use of AI tools is fair to other students.	Yapay zekâ kullanımımın diğer öğrenciler açısından adil olup olmadığını dikkate alırım.	Yes	No
	**a04**	I follow course rules about when and how AI tools may be used.	Yapay zekâ kullanımına ilişkin ders kurallarına uygun davranırım.	Yes	Yes
	**a05**	I avoid using AI tools to complete assessments that must be done independently.	Bireysel olarak tamamlamam gereken sınav, ödev veya değerlendirmelerde yapay zekâ kullanmaktan kaçınırım.	Yes	Yes
	**a06**	I make sure my final work reflects my own understanding, even when I use AI tools.	Yapay zekâdan yararlansam bile teslim ettiğim çalışmanın kendi anlayışımı yansıtmasına özen gösteririm.	Yes	Yes
	**a07**	I use AI tools in ways that do not violate academic integrity policies.	Yapay zekâyı akademik dürüstlük ilkelerine uygun biçimde kullanırım.	Yes	Yes
	**a08**	I avoid copying AI-generated text directly without proper revision or attribution.	Yapay zekânın ürettiği metni düzenlemeden ya da kullandığımı belirtmeden doğrudan kullanmamaya dikkat ederim.	Yes	Yes
	**a09**	I believe using AI tools is always acceptable if it helps me get a higher grade.	Daha yüksek not almamı sağlıyorsa yapay zekâ kullanmanın her durumda kabul edilebilir olduğunu düşünürüm.	No	
	**a10**	I think AI-generated work belongs to me as long as I submit it from my own account.	Kendi hesabımdan teslim ettiğim sürece yapay zekâ tarafından hazırlanmış bir çalışmanın bana ait olduğunu düşünürüm.	No	
	**a11**	I use AI tools without considering whether my classmates have the same access.	Diğer öğrencilerin aynı imkânlara sahip olup olmadığını düşünmeden yapay zekâ kullanırım.	No	
	**a12**	I rely on AI tools even when the instructor has not explained whether they are allowed.	Öğretmenim yapay zekâ kullanımına izin verilip verilmediğini açıklamamış olsa bile yapay zekâdan yararlanırım.	No	
**Monitor**	**b01**	I use AI tools to track whether my answers show enough understanding.	Cevaplarımın konuyu ne kadar anladığımı gösterip göstermediğini izlemek için yapay zekâdan yararlanırım.	Yes	Yes
	**b02**	I use AI tools to check whether I understand the main ideas of a topic.	Bir konunun temel fikirlerini anlayıp anlamadığımı kontrol etmek için yapay zekâdan yararlanırım.	Yes	Yes
	**b03**	I use AI tools to identify gaps in my understanding.	Anlayışımdaki eksikleri fark etmek için yapay zekâdan yararlanırım.	Yes	Yes
	**b04**	I use AI tools to compare my current understanding with what I am expected to know.	Mevcut bilgimi öğrenmem gereken bilgilerle karşılaştırmak için yapay zekâdan yararlanırım.	Yes	Yes
	**b05**	I use AI tools to notice when I am confused about a concept.	Bir kavramı karıştırıp karıştırmadığımı fark etmek için yapay zekâdan yararlanırım.	Yes	Yes
	**b06**	I use AI tools to review my progress while working on a learning task.	Bir öğrenme görevi üzerinde çalışırken ilerlememi gözden geçirmek için yapay zekâdan yararlanırım.	Yes	Yes
	**b07**	I use AI tools to test myself on what I have learned.	Öğrendiklerimi sınamak için yapay zekâdan yararlanırım.	Yes	No
	**b08**	I use AI tools to determine whether I need more practice on a topic.	Bir konuda daha fazla alıştırma yapmam gerekip gerekmediğini belirlemek için yapay zekâdan yararlanırım.	Yes	No
	**b09**	I know that I understand a topic whenever an AI explanation sounds simple.	Yapay zekânın açıklaması basit gelirse konuyu anladığımı düşünürüm.	No	
	**b10**	I use AI tools to tell me whether I studied enough without checking my own performance.	Kendi performansımı değerlendirmeden, yeterince çalışıp çalışmadığımı yapay zekânın söylemesine güvenirim.	No	
	**b11**	I ask AI tools to decide whether I am ready for an exam without answering practice questions.	Alıştırma veya deneme çözmeden sınava hazır olup olmadığıma yapay zekânın karar vermesini isterim.	No	
	**b12**	I assume that I have learned a topic if AI gives me a short explanation.	Yapay zekâ bir konuyu kısaca açıkladığında o konuyu öğrenmiş olduğumu varsayarım.	No	
**Motivate**	**c01**	I use AI tools to encourage myself when I feel unmotivated to study.	Ders çalışmaya isteksiz olduğumda kendimi motive etmek için yapay zekâdan yararlanırım.	Yes	Yes
	**c02**	I use AI tools to make boring academic tasks feel more interesting.	Sıkıcı bulduğum akademik görevleri daha ilgi çekici hâle getirmek için yapay zekâdan yararlanırım.	Yes	No
	**c03**	I use AI tools to remind myself why completing a learning task is important.	Bir öğrenme görevini tamamlamanın benim için neden önemli olduğunu hatırlamak için yapay zekâdan yararlanırım.	Yes	Yes
	**c04**	I use AI tools to maintain my effort when an assignment feels difficult.	Bir ödev zor geldiğinde çabamı sürdürmek için yapay zekâdan destek alırım.	Yes	Yes
	**c05**	I use AI tools to generate positive messages that help me continue studying.	Çalışmaya devam edebilmek için yapay zekâdan motive edici ifadeler isterim.	Yes	No

	**c06**	I use AI tools to connect learning tasks with my personal goals.	Öğrenme görevlerini kişisel hedeflerimle ilişkilendirmek için yapay zekâdan yararlanırım.	Yes	Yes
	**c07**	I use AI tools to help myself stay confident during challenging learning activities.	Zorlayıcı öğrenme etkinliklerinde özgüvenimi korumak için yapay zekâdan destek alırım.	Yes	Yes
	**c08**	I use AI tools to reduce my frustration when I struggle with academic work.	Akademik çalışmalarda zorlandığımda moralimi toparlamak için yapay zekâdan yararlanırım.	Yes	Yes
	**c09**	I use AI tools so that I do not have to push myself during difficult assignments.	Zor ödevlerde fazla çaba göstermemek için yapay zekâya başvururum.	No	
	**c10**	I feel motivated only when AI tools give me direct praise.	Ancak yapay zekâ beni açıkça övdüğünde motive olurum.	No	
	**c11**	I use AI tools to avoid academic tasks that make me uncomfortable.	Beni zorlayan akademik görevlerden kaçınmak için yapay zekâ kullanırım.	No	
	**c12**	I ask AI tools to make me feel successful even when I have not worked on the task.	Görev üzerinde yeterince çalışmamış olsam bile kendimi başarılı hissetmek için yapay zekâdan destek isterim.	No	
**Regulate**	**d01**	I use AI tools to identify the resources I need before starting an assignment.	Bir ödeve başlamadan önce ihtiyaç duyacağım kaynakları belirlemek için yapay zekâdan yararlanırım.	Yes	Yes
	**d02**	I use AI tools to organize my study tasks before I begin learning.	Çalışmaya başlamadan önce yapmam gerekenleri düzenlemek için yapay zekâdan yararlanırım.	Yes	Yes
	**d03**	I use AI tools to divide large assignments into smaller, manageable steps.	Büyük ödevleri daha küçük ve yönetilebilir adımlara ayırmak için yapay zekâdan yararlanırım.	Yes	Yes
	**d04**	I use AI tools to decide which parts of a task I should complete first.	Bir göreve nereden başlamam gerektiğine karar vermek için yapay zekâdan yararlanırım.	Yes	Yes
	**d05**	I use AI tools to plan how much time I should spend on different learning activities.	Farklı öğrenme etkinliklerine ne kadar zaman ayıracağımı planlamak için yapay zekâdan yararlanırım.	Yes	Yes
	**d06**	I use AI tools to create a learning schedule for completing academic tasks.	Akademik görevlerimi tamamlamak için çalışma takvimi hazırlarken yapay zekâdan yararlanırım.	Yes	Yes
	**d07**	I use AI tools to set specific learning goals for my coursework.	Derslerim için belirli öğrenme hedefleri oluştururken yapay zekâdan yararlanırım.	Yes	No
	**d08**	I use AI tools to adjust my study plan when I fall behind.	Çalışma planımın gerisinde kaldığımda planımı yeniden düzenlemek için yapay zekâdan yararlanırım.	Yes	No
	**d09**	I let AI tools decide my learning goals without thinking about my course requirements.	Dersin gereklerini dikkate almadan öğrenme hedeflerimi yapay zekânın belirlemesine izin veririm.	No	
	**d10**	I use AI tools to plan my study time without considering deadlines.	Teslim tarihlerini dikkate almadan çalışma zamanımı yapay zekâ ile planlarım.	No	
	**d11**	I ask AI tools to create a plan that allows me to finish all work at the last minute.	Tüm işlerimi son dakikaya bırakabileceğim bir plan hazırlaması için yapay zekâdan yardım isterim.	No	
	**d12**	I follow an AI-generated study plan even when it does not match my learning needs.	Öğrenme ihtiyaçlarıma uygun olmasa bile yapay zekânın hazırladığı çalışma planını aynen uygularım.	No	
**Evaluate**	**e01**	I verify important facts provided by AI tools before including them in my work.	Yapay zekânın verdiği önemli bilgileri çalışmama eklemeden önce doğrularım.	Yes	Yes
	**e02**	I judge whether an AI-generated answer is relevant to the task requirements.	Yapay zekânın verdiği bir cevabın görevle ne kadar ilgili olduğunu değerlendiririm.	Yes	Yes
	**e03**	I check whether AI-generated information is accurate before using it.	Yapay zekânın verdiği bilgileri kullanmadan önce doğruluğunu kontrol ederim.	Yes	Yes
	**e04**	I compare AI-generated answers with reliable academic sources.	Yapay zekânın verdiği cevapları güvenilir akademik kaynaklarla karşılaştırırım.	Yes	Yes
	**e05**	I examine whether AI-generated explanations are supported by evidence.	Yapay zekânın yaptığı açıklamaların kanıtlara dayanıp dayanmadığını incelerim.	Yes	No
	**e06**	I look for errors or misleading claims in AI-generated content.	Yapay zekânın oluşturduğu içerikte hata veya yanıltıcı ifade olup olmadığını kontrol ederim.	Yes	No
	**e07**	I question AI-generated information instead of accepting it immediately.	Yapay zekânın verdiği bilgileri hemen kabul etmek yerine sorgularım.	Yes	Yes
	**e08**	I decide whether AI-generated suggestions are appropriate for the academic context.	Yapay zekânın önerilerinin akademik bağlama uygun olup olmadığını değerlendiririm.	Yes	Yes
	**e09**	I trust AI-generated information when it is written in a professional tone.	Profesyonel bir dille yazılmışsa yapay zekânın verdiği bilgilere güvenirim.	No	
	**e10**	I accept AI-generated citations if they look complete.	Eksiksiz görünüyorsa yapay zekânın oluşturduğu kaynakçaları doğru kabul ederim.	No	
	**e11**	I use AI-generated answers without checking whether they match the course content.	Ders içeriğiyle uyumlu olup olmadığını kontrol etmeden yapay zekânın verdiği cevapları kullanırım.	No	
	**e12**	I assume AI-generated explanations are reliable because AI tools process large amounts of information.	Yapay zekâ çok fazla bilgiyle çalıştığı için yaptığı açıklamaların güvenilir olduğunu varsayarım.	No	

Appendix C

AI-Assisted Learning Judgment Quiz: Items, Response Options, and Answer Key

Construct	Quiz item	Field	English	Turkish	Correct answer
Ethical Use	1	Scenario	A student uses an AI tool to generate several ideas for a history essay. The student writes the final essay independently, changes the structure, adds course readings, and follows the instructor's requirement to disclose AI use.	Bir öğrenci tarih dersi için yazacağı kompozisyonda yapay zekâdan birkaç fikir alır. Son metni kendisi yazar, metnin yapısını değiştirir, derste kullanılan kaynakları ekler ve öğretmeninin istediği şekilde yapay zekâ kullandığını belirtir.	
		Question	What is the best judgment of the student's action?	Bu öğrencinin yaptığı en doğru şekilde nasıl değerlendirilir?	
		Answer Choice 1	The student acted unethically because AI was used at any stage.	Öğrenci yapay zekâ kullandığı için her durumda yanlış davranmıştır.	
		Answer Choice 2	The student should submit the AI-generated ideas without changes.	Öğrenci yapay zekânın verdiği fikirleri değiştirmeden teslim etmelidir.	
		Answer Choice 3	The student used AI appropriately because the final work reflects personal understanding and AI use was disclosed.	Öğrenci yapay zekâyı uygun şekilde kullanmıştır; çünkü son çalışma kendi anlayışını yansıtmaktadır ve yapay zekâ kullandığını belirtmiştir.	**Correct**
		Answer Choice 4	The student should hide the AI use because the final essay was written independently.	Son metni kendisi yazdığı için yapay zekâ kullandığını söylemesine gerek yoktur.	
		Answer Choice 5	The student should only cite the AI tool and remove all course readings.	Öğrenci yalnızca yapay zekâyı kaynak göstermeli, ders kaynaklarını çıkarmalıdır.	
	2	Scenario	An instructor states that AI may be used for brainstorming but not for writing final answers. A student asks an AI tool to write the full response and submits it with only minor wording changes.	Öğretmen, yapay zekânın yalnızca fikir üretmek için kullanılabileceğini, ancak son cevapların yapay zekâya yazdırılamayacağını söyler. Bir öğrenci cevabın tamamını yapay zekâya yazdırır ve yalnızca birkaç kelimeyi değiştirerek teslim eder.	
		Question	What is the best judgment of this behavior?	Bu davranış en doğru şekilde nasıl değerlendirilir?	
		Answer Choice 1	It is acceptable because the student changed a few words.	Öğrenci birkaç kelimeyi değiştirdiği için bu kabul edilebilir.	
		Answer Choice 2	It is acceptable because the AI response was related to the assignment.	Yapay zekânın cevabı ödevle ilgili olduğu için bu kabul edilebilir.	
		Answer Choice 3	It is inappropriate because the student violated the instructor's rule and submitted AI-generated work as personal work.	Bu uygun değildir; çünkü öğrenci öğretmenin kuralını ihlal etmiş ve yapay zekânın yazdığı cevabı kendi çalışması gibi teslim etmiştir.	**Correct**
		Answer Choice 4	It is appropriate as long as the student understood the topic afterward.	Öğrenci konuyu sonradan anladıysa bu uygundur.	
		Answer Choice 5	It is appropriate because AI tools are commonly used in education.	Yapay zekâ eğitimde yaygın kullanıldığı için bu uygundur.	
	3	Scenario	A student is completing an online quiz that must be completed independently. The student is unsure about one question and considers copying the question into an AI tool to get the answer.	Bir öğrenci tek başına tamamlaması gereken çevrim içi bir kısa sınav yapmaktadır. Bir sorudan emin olamaz ve cevabı bulmak için soruyu yapay zekâya yazmayı düşünür.	
		Question	What should the student do?	Öğrenci ne yapmalıdır?	
		Answer Choice 1	Use AI only for the difficult question because the rest of the quiz is independent work.	Sadece zorlandığı soru için yapay zekâ kullanmalıdır; çünkü diğer soruları kendisi çözmektedir.	
		Answer Choice 2	Use AI and then rewrite the answer in different words.	Yapay zekâdan cevap alıp cevabı kendi cümleleriyle yeniden yazmalıdır.	
		Answer Choice 3	Ask AI for the answer but avoid copying it directly.	Cevabı yapay zekâya sormalı ama doğrudan kopyalamamalıdır.	
		Answer Choice 4	Avoid using AI because the assessment is required to be completed independently.	Değerlendirmenin bireysel olarak yapılması gerektiği için yapay zekâ kullanmamalıdır.	**Correct**
		Answer Choice 5	Use AI after submitting the quiz to check whether the answer was correct.	Sınavı teslim ettikten sonra cevabını kontrol etmek için yapay zekâ kullanmalıdır.	
Monitor	1	Scenario	A student asks an AI tool to explain a biology concept. The explanation seems clear, but the student is not sure whether they truly understand it.	Bir öğrenci biyolojiyle ilgili bir kavramı yapay zekâya açıklatır. Açıklama anlaşılır görünür, ancak öğrenci konuyu gerçekten anlayıp anlamadığından emin değildir.	
		Question	What is the best next step?	Bundan sonra atılacak en doğru adım nedir?	
		Answer Choice 1	Ask the AI tool to give practice questions and try answering them without help.	Yapay zekâdan alıştırma soruları istemeli ve bu soruları yardım almadan çözmeye çalışmalıdır.	**Correct**
		Answer Choice 2	Copy the explanation into notes because it sounds clear.	Açıklama anlaşılır göründüğü için notlarına aynen geçirmelidir.	
		Answer Choice 3	Move to the next topic because the AI explanation was easy to read.	Yapay zekânın açıklaması kolay okunuyorsa bir sonraki konuya geçmelidir.	
		Answer Choice 4	Ask the AI tool to make the explanation shorter.	Yapay zekâdan açıklamayı daha kısa yazmasını istemelidir.	
		Answer Choice 5	Assume understanding is complete because the concept now feels familiar.	Kavram artık tanıdık geldiği için konuyu tamamen anladığını varsaymalıdır.	

	2	Scenario	A student is studying for an exam with an AI tutor. After reading several AI explanations, the student wants to identify which parts of the topic are still weak.	Bir öğrenci sınava çalışırken yapay zekâdan ders anlatımı desteği alır. Birkaç açıklama okuduktan sonra konunun hangi kısımlarında hâlâ eksikleri olduğunu görmek ister.	
		Question	What is the best prompt to use?	Yapay zekâya hangisini sorması en uygundur?	
		Answer Choice 1	“Give me a perfect summary of this topic.”	“Bu konunun kusursuz bir özetini çıkar.”	
		Answer Choice 2	“Write my exam answers for this topic.”	“Bu konuyla ilgili sınav cevaplarımı yaz.”	
		Answer Choice 3	“Make this topic sound easier.”	“Bu konuyu daha kolaymış gibi anlat.”	
		Answer Choice 4	“Tell me the most common facts about this topic.”	“Bu konuyla ilgili en yaygın bilgileri söyle.”	
		Answer Choice 5	“Ask me diagnostic questions and identify which subtopics I misunderstand.”	“Bana konuyu yoklayacak sorular sor ve hangi alt konuları yanlış ya da eksik anladığımı belirle.”	**Correct**
	3	Scenario	A student answers an AI-generated practice question incorrectly. The AI provides the correct answer immediately. The student wants to monitor their learning rather than simply receive the solution.	Bir öğrenci yapay zekânın hazırladığı bir alıştırma sorusunu yanlış cevaplar. Yapay zekâ doğru cevabı hemen verir. Öğrenci yalnızca cevabı almak yerine kendi öğrenmesini kontrol etmek ister.	
		Question	What should the student do?	Öğrenci ne yapmalıdır?	
		Answer Choice 1	Memorize the correct answer and continue.	Doğru cevabı ezberleyip devam etmelidir.	
		Answer Choice 2	Ask the AI to make the answer shorter.	Yapay zekâdan cevabı daha kısa yazmasını istemelidir.	
		Answer Choice 3	Ask the AI to explain the mistake and then try a similar problem independently.	Yapay zekâdan hatasını açıklamasını istemeli ve ardından benzer bir soruyu kendi başına çözmeye çalışmalıdır.	**Correct**
		Answer Choice 4	Skip the topic because one mistake does not matter.	Tek bir hata önemli olmadığı için konuyu geçmelidir.	
		Answer Choice 5	Copy the explanation into the assignment.	Açıklamayı ödevine aynen eklemelidir.	
Motivate	1	Scenario	A student feels discouraged while working on a long research assignment and is considering giving up for the day. The student wants to use AI to support motivation without avoiding the task.	Bir öğrenci uzun bir araştırma ödevi üzerinde çalışırken hevesini kaybeder ve o gün çalışmayı bırakmayı düşünür. Öğrenci görevden kaçmadan motivasyonunu desteklemek için yapay zekâ kullanmak ister.	
		Question	What is the best use of AI?	Yapay zekâyı en uygun kullanma biçimi hangisidir?	
		Answer Choice 1	Ask AI to break the task into small achievable steps and suggest a short first action.	Yapay zekâdan görevi küçük ve yapılabilir adımlara ayırmasını ve hemen başlanabilecek kısa bir ilk adım önermesini istemek.	**Correct**
		Answer Choice 2	Ask AI to complete the full assignment.	Yapay zekâdan tüm ödevi tamamlamasını istemek.	
		Answer Choice 3	Ask AI to explain why the assignment is not important.	Yapay zekâdan ödevin neden önemli olmadığını açıklamasını istemek.	
		Answer Choice 4	Ask AI to replace the assignment with an easier topic.	Yapay zekâdan ödevi daha kolay bir konuyla değiştirmesini istemek.	
		Answer Choice 5	Ask AI for unrelated entertainment until motivation returns.	Motivasyonu gelene kadar yapay zekâdan konuyla ilgisiz eğlenceli içerikler istemek.	
	2	Scenario	A student finds a required reading boring and is losing interest. The student wants to use AI to increase engagement while still learning the assigned material.	Bir öğrenci okuması gereken metni sıkıcı bulur ve ilgisi azalır. Yine de verilen metni öğrenerek ilerlemek için yapay zekâdan yararlanmak ister.	
		Question	What is the best strategy?	En uygun strateji hangisidir?	
		Answer Choice 1	Ask AI to remove the difficult parts of the reading.	Yapay zekâdan metindeki zor kısımları çıkarmasını istemek.	
		Answer Choice 2	Ask AI to write a response without reading the text.	Metni okumadan cevap yazması için yapay zekâdan yardım istemek.	
		Answer Choice 3	Ask AI to summarize only the easiest paragraph.	Yapay zekâdan yalnızca en kolay paragrafı özetlemesini istemek.	
		Answer Choice 4	Ask AI to connect the reading to the student’s interests and generate guiding questions.	Yapay zekâdan metni öğrencinin ilgi alanlarıyla ilişkilendirmesini ve okurken yol gösterecek sorular hazırlamasını istemek.	**Correct**
		Answer Choice 5	Ask AI to tell the student that the reading is unnecessary.	Yapay zekâdan metni okumanın gereksiz olduğunu söylemesini istemek.	
	3	Scenario	A student feels anxious before starting a difficult statistics problem set. The student wants to use AI to maintain confidence and persistence.	Bir öğrenci zor bir istatistik problem setine başlamadan önce kaygılanır. Özgüvenini korumak ve çalışmayı sürdürmek için yapay zekâdan yararlanmak ister.	
		Question	What is the best approach?	En uygun yaklaşım hangisidir?	
		Answer Choice 1	Ask AI to give only the final answers.	Yapay zekâdan yalnızca son cevapları istemek.	

		Answer Choice 2	Ask AI to confirm that the student does not need to understand statistics.	Yapay zekâdan istatistiği anlamasına gerek olmadığını söylemesini istemek.	
		Answer Choice 3	Ask AI to rewrite the problems so they are less demanding.	Yapay zekâdan soruları daha kolay hâle getirmesini istemek.	
		Answer Choice 4	Ask AI to tell the student that mistakes do not matter.	Yapay zekâdan hataların hiç önemli olmadığını söylemesini istemek.	
		Answer Choice 5	Ask AI to provide encouragement, a step-by-step practice plan, and reminders to check progress.	Yapay zekâdan cesaret verici destek, adım adım bir çalışma planı ve ilerlemeyi kontrol etmeye yönelik hatırlatmalar istemek.	**Correct**
Regulate	1	Scenario	A student has three assignments due in the same week and wants to use AI to manage the workload.	Bir öğrencinin aynı hafta teslim etmesi gereken üç ödevi vardır. Öğrenci iş yükünü planlamak için yapay zekâdan yararlanmak ister.	
		Question	What is the best AI-supported regulation strategy?	Yapay zekâ destekli en uygun düzenleme stratejisi hangisidir?	
		Answer Choice 1	Ask AI to complete the shortest assignment first.	Yapay zekâdan önce en kısa ödevi tamamlamasını istemek.	
		Answer Choice 2	Ask AI to help create a schedule based on deadlines, task difficulty, and available study time.	Teslim tarihlerini, ödevlerin zorluk düzeyini ve çalışma için ayrılabilecek zamanı dikkate alan bir plan hazırlamak için yapay zekâdan yardım istemek.	**Correct**
		Answer Choice 3	Ask AI to choose one assignment to ignore.	Yapay zekâdan hangi ödevi görmezden gelmesi gerektiğini seçmesini istemek.	
		Answer Choice 4	Ask AI to write all three assignments.	Üç ödevi de yapay zekâya yazdırmak.	
		Answer Choice 5	Ask AI to make the deadlines seem less stressful.	Yapay zekâdan teslim tarihlerini daha az stresli göstermesini istemek.	
	2	Scenario	A student begins a large project but does not know where to start. The student wants to regulate the learning process effectively.	Bir öğrenci büyük bir projeye başlayacaktır, ancak nereden başlayacağını bilemez. Öğrenci öğrenme sürecini daha düzenli yürütmek ister.	
		Question	What should the student ask AI to do?	Öğrenci yapay zekâdan ne istemelidir?	
		Answer Choice 1	Help divide the project into stages with specific goals for each stage.	Projeyi aşamalara ayırmasına ve her aşama için belirli hedefler koymasına yardım etmesini istemelidir.	**Correct**
		Answer Choice 2	Produce the final project immediately.	Projenin tamamını hemen hazırlamasını istemelidir.	
		Answer Choice 3	Suggest unrelated easier topics.	Konuyla ilgisiz, daha kolay başlıklar önermesini istemelidir.	
		Answer Choice 4	Predict the grade without seeing the work.	Çalışmayı görmeden alacağı notu tahmin etmesini istemelidir.	
		Answer Choice 5	Replace the project requirements with simpler ones.	Proje gerekliliklerini daha kolay olanlarla değiştirmesini istemelidir.	
	3	Scenario	A student planned to study two chapters in one evening but realizes after one hour that the plan is unrealistic. The student wants to use AI appropriately.	Bir öğrenci bir akşamda iki ünite çalışmayı planlar, ancak bir saat sonra bu planın gerçekçi olmadığını fark eder. Öğrenci yapay zekâyı uygun şekilde kullanmak ister.	
		Question	What is the best next step?	Bundan sonra atılacak en doğru adım nedir?	
		Answer Choice 1	Continue the original plan even if learning becomes superficial.	Öğrenme yüzeysel kalsa bile ilk plana aynen devam etmek.	
		Answer Choice 2	Stop studying completely because the plan failed.	Plan tutmadığı için çalışmayı tamamen bırakmak.	
		Answer Choice 3	Ask AI to help revise the plan by prioritizing key sections and rescheduling unfinished work.	Önemli kısımları önceliklendirmek ve bitmeyen çalışmayı yeniden planlamak için yapay zekâdan yardım istemek.	**Correct**
		Answer Choice 4	Ask AI to summarize both chapters so the student can avoid studying.	Çalışmamak için iki üniteyi de yapay zekâya özetletmek.	
		Answer Choice 5	Ignore the problem and hope there is enough time later.	Sorunu görmezden gelip sonra yeterli zaman kalacağını ummak.	
Evaluate	1	Scenario	An AI tool gives a surprising claim about climate change for a science assignment. The claim sounds sophisticated, but the student has not seen it in class materials.	Bir öğrenci fen dersi ödevi için yapay zekâdan iklim değişikliğiyle ilgili şaşırtıcı bir bilgi alır. Bilgi kulağa bilimsel gelir, ancak öğrenci bu bilgiyi ders materyallerinde görmemiştir.	
		Question	What should the student do before using the claim?	Öğrenci bu bilgiyi kullanmadan önce ne yapmalıdır?	
		Answer Choice 1	Use it because sophisticated language usually means the information is reliable.	Bilimsel bir dille yazıldığı için bilgiyi kullanmalıdır.	
		Answer Choice 2	Use it if the AI gives the same answer twice.	Yapay zekâ aynı cevabı iki kez verirse bilgiyi kullanmalıdır.	
		Answer Choice 3	Use it only if it makes the assignment more interesting.	Ödevi daha ilgi çekici hâle getiriyorsa bilgiyi kullanmalıdır.	
		Answer Choice 4	Use it because AI tools are trained on large amounts of information.	Yapay zekâ çok fazla bilgiyle eğitildiği için bilgiyi kullanmalıdır.	
		Answer Choice 5	Check the claim against reliable scientific or academic sources.	Bu bilgiyi güvenilir bilimsel veya akademik kaynaklardan kontrol etmelidir.	**Correct**

	2	Scenario	A student asks AI for sources for a psychology paper. The AI provides several citations, but the student cannot find one of the articles in the library database or through a search engine.	Bir öğrenci psikoloji ödevi için yapay zekâdan kaynak önerileri ister. Yapay zekâ birkaç kaynakça bilgisi verir, ancak öğrenci makalelerden birini ne kütüphane veri tabanında ne de internette bulabilir.	
		Question	What is the best judgment?	Bu durumda en doğru değerlendirme hangisidir?	
		Answer Choice 1	The citation should be used because AI-generated references are usually accurate.	Yapay zekânın verdiği kaynakçalar genellikle doğru olduğu için bu kaynak kullanılmalıdır.	
		Answer Choice 2	The citation should be used if the title sounds academic.	Başlık akademik görünüyorsa kaynak kullanılmalıdır.	
		Answer Choice 3	The student should invent missing details to complete the reference.	Öğrenci eksik bilgileri kendisi tamamlayarak kaynağı kullanmalıdır.	
		Answer Choice 4	The student should treat the citation as unreliable unless it can be verified.	Doğrulanamadığı sürece bu kaynak güvenilir kabul edilmemelidir.	**Correct**
		Answer Choice 5	The student should cite the AI instead of checking the source.	Kaynağı kontrol etmek yerine yapay zekâyı kaynak göstermelidir.	
	3	Scenario	A student asks AI to explain a mathematical procedure. The explanation gives the correct final answer, but one step appears inconsistent with the formula taught in class.	Bir öğrenci matematikteki bir çözüm yöntemini yapay zekâya açıklatır. Açıklamada son cevap doğru görünür, ancak adımlardan biri derste öğrenilen formülle uyuşmaz.	
		Question	What should the student do?	Öğrenci ne yapmalıdır?	
		Answer Choice 1	Accept the explanation because the final answer is correct.	Son cevap doğru olduğu için açıklamayı kabul etmelidir.	
		Answer Choice 2	Compare the AI's steps with class notes or another reliable source before using the explanation.	Açıklamayı kullanmadan önce yapay zekânın çözüm adımlarını ders notlarıyla veya başka güvenilir bir kaynakla karşılaştırmalıdır.	**Correct**
		Answer Choice 3	Delete the confusing step and keep the rest without checking.	Kafasını karıştıran adımı silip kalan kısmı kontrol etmeden kullanmalıdır.	
		Answer Choice 4	Assume the class formula is outdated.	Derste öğrenilen formülün artık geçerli olmadığını varsaymalıdır.	
		Answer Choice 5	Use the AI explanation because it is faster than checking.	Kontrol etmek zaman alacağı için yapay zekânın açıklamasını kullanmalıdır.	

Appendix D

Complete Standardized EFA Pattern Matrix and Uniqueness Values

Item	Factor 1	Factor 2	Factor 3	Factor 4	Factor 5	Uniqueness
a1	-0.015	**0.796**	0.018	0.000	0.055	0.367
a4	0.073	**0.808**	0.024	-0.013	-0.080	0.336
a5	-0.035	**0.786**	0.009	0.004	0.009	0.379
a6	-0.030	**0.767**	-0.017	0.028	-0.017	0.404
a7	-0.047	**0.752**	-0.001	0.002	-0.016	0.426
a8	0.047	**0.794**	-0.009	-0.015	0.043	0.375
b1	-0.144	-0.018	0.013	**0.804**	0.065	0.347
b2	0.098	0.034	-0.041	**0.762**	-0.016	0.400
b3	-0.010	-0.037	0.075	**0.704**	-0.022	0.488
b4	0.004	0.055	-0.037	**0.720**	-0.031	0.490
b5	0.056	0.024	-0.037	**0.716**	0.040	0.466
b6	-0.025	-0.053	0.003	**0.783**	-0.043	0.406
d1	0.011	-0.022	**0.792**	-0.045	-0.088	0.398
d2	0.042	-0.002	**0.702**	-0.002	0.024	0.481
d3	-0.021	0.015	**0.807**	0.014	0.013	0.348
d4	-0.087	0.039	**0.787**	-0.045	0.011	0.417
d5	0.081	-0.029	**0.646**	0.055	0.055	0.508
d6	-0.016	0.016	**0.757**	0.008	-0.016	0.434
e1	**0.792**	0.043	-0.055	0.031	0.042	0.372
e2	**0.819**	-0.032	-0.013	-0.027	0.029	0.327
e3	**0.817**	-0.036	-0.051	-0.017	-0.041	0.364
e4	**0.675**	-0.010	0.074	0.089	-0.005	0.482
e7	**0.760**	-0.006	0.032	0.001	-0.022	0.414
e8	**0.829**	0.031	0.016	-0.089	0.006	0.323
c1	0.080	0.053	-0.005	-0.015	**0.725**	0.452
c3	-0.021	-0.060	0.056	0.022	**0.639**	0.565
c4	-0.013	0.056	-0.039	-0.036	**0.817**	0.361
c6	-0.006	0.009	0.048	0.062	**0.682**	0.500
c7	-0.030	-0.027	0.007	-0.027	**0.817**	0.344
c8	-0.002	-0.023	-0.070	-0.020	**0.693**	0.536

Note. All factor loadings are displayed without suppression. Factors were extracted using principal axis factoring with Promax rotation. Uniqueness values, rather than communalities, are reported. Factor 1 = Evaluate; Factor 2 = Ethical Use; Factor 3 = Regulate; Factor 4 = Monitor; Factor 5 = Motivate.
